# Tavaxy: Integrating Taverna and Galaxy workflows with cloud computing support

**DOI:** 10.1186/1471-2105-13-77

**Published:** 2012-05-04

**Authors:** Mohamed Abouelhoda, Shadi Alaa Issa, Moustafa Ghanem

**Affiliations:** 1 Center for Informatics Sciences, Nile University, Giza, Egypt; 2 Department of Computing, Imperial College London, London, SW7 2AZ, UK; 3 Faculty of Engineering, Cairo University, Giza, Egypt

## Abstract

**Background:**

Over the past decade the workflow system paradigm has evolved as an efficient and user-friendly approach for developing complex bioinformatics applications. Two popular workflow systems that have gained acceptance by the bioinformatics community are Taverna and Galaxy. Each system has a large user-base and supports an ever-growing repository of application workflows. However, workflows developed for one system cannot be imported and executed easily on the other. The lack of interoperability is due to differences in the models of computation, workflow languages, and architectures of both systems. This lack of interoperability limits sharing of workflows between the user communities and leads to duplication of development efforts.

**Results:**

In this paper, we present *Tavaxy*, a stand-alone system for creating and executing workflows based on using an extensible set of re-usable workflow patterns. *Tavaxy *offers a set of new features that simplify and enhance the development of sequence analysis applications: It allows the integration of existing Taverna and Galaxy workflows in a single environment, and supports the use of cloud computing capabilities. The integration of existing Taverna and Galaxy workflows is supported seamlessly at both run-time and design-time levels, based on the concepts of hierarchical workflows and workflow patterns. The use of cloud computing in *Tavaxy *is flexible, where the users can either instantiate the whole system on the cloud, or delegate the execution of certain sub-workflows to the cloud infrastructure.

**Conclusions:**

*Tavaxy *reduces the workflow development cycle by introducing the use of workflow patterns to simplify workflow creation. It enables the re-use and integration of existing (sub-) workflows from Taverna and Galaxy, and allows the creation of hybrid workflows. Its additional features exploit recent advances in high performance cloud computing to cope with the increasing data size and complexity of analysis.

The system can be accessed either through a cloud-enabled web-interface or downloaded and installed to run within the user's local environment. All resources related to *Tavaxy *are available at http://www.tavaxy.org.

## Background

### Increasing complexity of analysis and scientific workflow paradigm

The advent of high-throughput sequencing technologies - accompanied with the recent advances in open source software tools, open access data sources, and cloud computing platforms - has enabled the genomics community to develop and use sophisticated application *workflows*. Such workflows start with voluminous raw sequences and end with detailed structural, functional, and evolutionary results. The workflows involve the use of multiple software tools and data resources in a staged fashion, with the output of one tool being passed as input to the next. As one example, a personalized medicine workflow [1-3] based on *Next Generation Sequencing *(NGS) technology can start with short DNA sequences (reads) of an individual human genome and end with a diagnostic and prognostic report, or potentially even with a treatment plan if clinical data were available. This workflow involves the use of multiple software tools to assess the quality of the reads, to map them to a reference human genome, to identify the sequence variations, to query databases for the sake of associating variations to diseases, and to check for novel variants. As another example, consider a workflow in the area of metagenomics [4-7]. Such workflow can start with a large collection of sequenced reads and end up with determination of the existing micro-organisms in the environmental sample and an estimation of their relative abundance. This workflow also involves different tasks and software tools, such as those used for assessing the quality of the reads, assembling them into longer DNA segments, querying them against different databases, and conducting phylogenetic and taxonomical analyses.

To simplify the design and execution of complex bioinformatics workflows, especially those that use multiple software tools and data resources, a number of scientific workflow systems have been developed over the past decade. Examples include Taverna [8,9], Kepler [10], Triana [11,12], Galaxy [13], Conveyor [14] Pegasus [15], Pegasys [16], Gene Pattern [17,18], Discovery Net [19,20], and OMII-BPEL [21]; see [22] for a survey and comparison of some of these tools.

All such workflow systems typically adopt an abstract representation of a workflow in the form of a directed graph, where nodes represent tasks to be executed and edges represent either data flow or execution dependencies between different tasks. Based on this abstraction, and through a visual front-end, the user can intuitively build and modify complex applications with little or no programming expertise. The workflow system maps the edges and nodes in the graph to real data and software components. The *workflow engine *(also called *execution *or *enactment engine*) executes the software components either locally on the user machine or remotely at distributed locations. The engine takes care of data transfer between the nodes and can also exploit the use of high performance computing architectures, if available, so that independent tasks run in parallel. This makes the application scientist focus on the logic of their applications and no longer worry about the technical details of invoking the software components or use of distributed computing resources.

Within the bioinformatics community, two workflow systems have gained increasing popularity, as reflected by their large and growing user communities. These are Galaxy [13] and Taverna [8,9]. Both systems are efficient, open source, and satisfy to a great extent the requirements of the bioinformatics community. Taverna has been developed primarily to simplify the development of workflows that access and use analyses tasks deployed as remote web and grid services. It comes with an associated directory of popular remote bioinformatics services and provides an environment that coordinates their invocation and execution. Galaxy has been developed primarily to facilitate the execution of software tools on local (high performance computing) infrastructure while still simplifying access to data held on remote biological resources. Its installation includes a large library of tools and pre-made scripts for data processing. Both systems are extensible, allowing their users to integrate new services and tools easily. Each system offers log files to capture the history of experiment details. Furthermore, both systems provide web-portals allowing users to share and publish their workflows: These are the myExperiment portal for Taverna [23,24] and the *Public Pages *for Galaxy [13]. The features of both systems are continuously being updated by their development teams and their user communities are active in developing and sharing new application workflows.

However, since both Taverna and Galaxy have been developed with different use cases and execution environments in mind, each system tends to be suited to different styles of bioinformatics applications. The key differences between the two systems can be categorized into three major classes:

1. Execution environment and system design: Taverna is oriented towards using web-services for invoking remote applications, while Galaxy is oriented towards efficient execution on a local infrastructures.

2. Model of computation: Taverna includes control constructs such as conditionals and iterations, and data constructs that can handle (nested) lists (in parallel) using a number of pre-defined operations. These constructs are not directly available in Galaxy, which puts a limitation on the types of workflows that can be executed on Galaxy.

3. Workflow description language: Taverna uses the XML-based language SCUFL for describing the workflows, while Galaxy expresses workflows in its own language using JSON format.

These differences lead to two major consequences: First, some tasks can be implemented easily on one system but would be difficult to implement on the other without considerable programming effort. Second, a (sub-) workflow developed on one system cannot be imported and re-used by the other easily (i.e., *lack of interoperability*), which limits sharing of workflows between their communities and leads to duplication of development efforts.

### Our contribution

**Figure 1:**
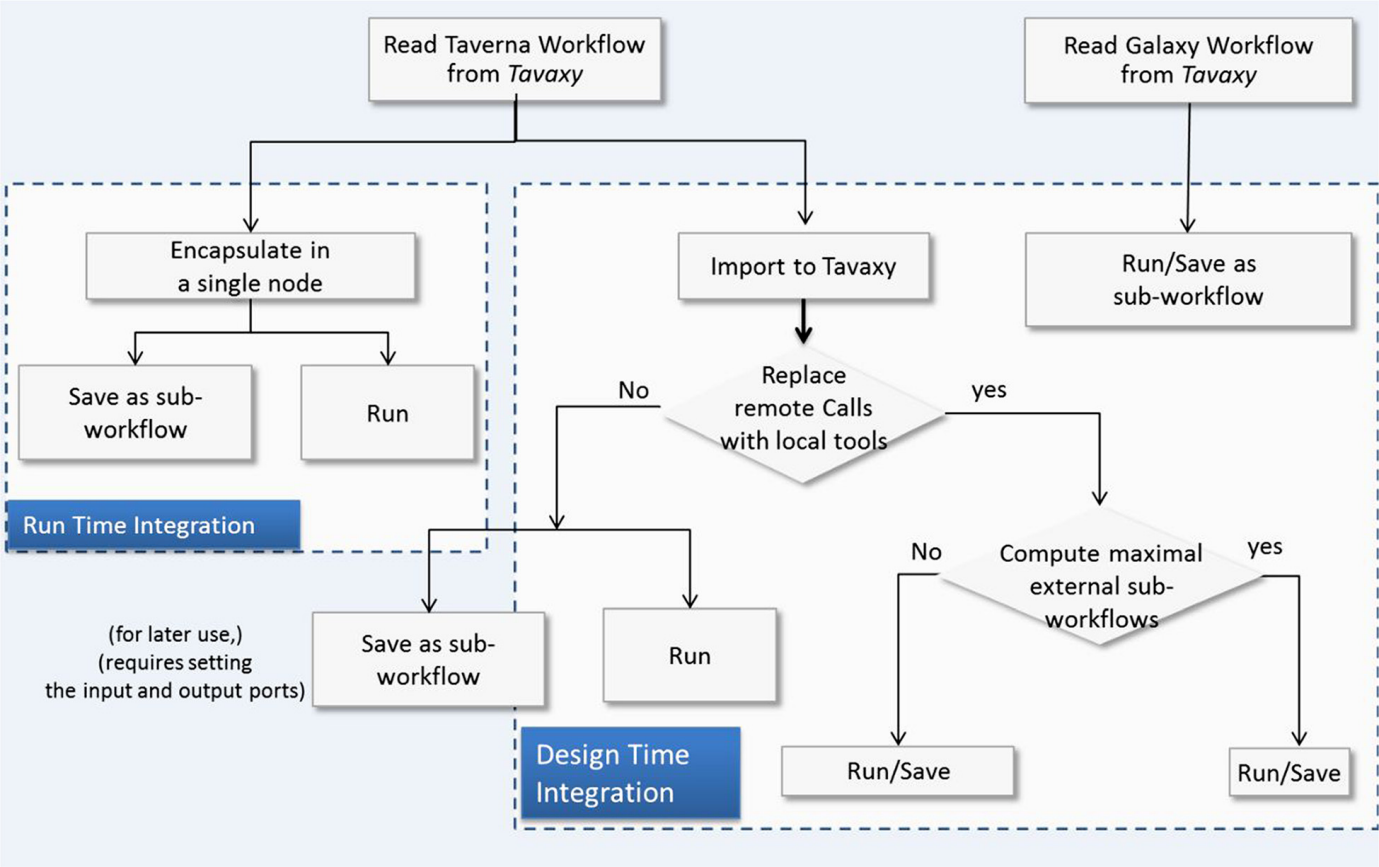
**Use diagram of integrating Taverna, Galaxy, and Tavaxy workflows.** Tavaxy is a standalone workflow system that executes Tavaxy workflows as well as integrates and executes Taverna and Galaxy workflows. Galaxy workflows are compatible with Tavaxy and can be imported and executed directly on the system. For Taverna workflows, the integration can take place at either run-time or design-time. At run time, the Taverna (sub-) workflows can be executed as a whole by calling the Taverna engine. They can also be saved as sub-workflows and used within other Tavaxy workflows. At workflow design time, Taverna workflows are translated to the Tavaxy language, enabling them to be edited and enhanced. In this case, the user has the option of replacing any of the remote calls in the Taverna workflow with calls to equivalent local tools. Any remaining Taverna sub-workflow fragments can be directly executed using the Taverna engine. As an optimization, sub-workflows can be encapsulated into maximal external sub-workflows so as to minimize execution overheads. The implementation section addresses the maximal external sub-workflows in more details.

In this paper, we present *Tavaxy*, a pattern-based workflow system that can integrate the use and execution of Taverna and Galaxy workflows in a single environment. The focus of *Tavaxy *is facilitating the efficient execution of sequence analysis tasks on high performance computing infrastructures and cloud computing systems. *Tavaxy *builds on the features of Taverna or Galaxy providing the following benefits:

· *Single entry point: Tavaxy *is a standalone pattern-based workflow system providing an extensible set of patterns, and allows easy integration with other workflow systems. It provides a single environment to open, edit, and execute its own workflows as well as integrate native Taverna and Galaxy whole- or sub-workflows, thus enabling users to compose hybrid workflows. Figure 1 summarizes the different integration use cases at run-time and design-time levels in *Tavaxy*. (The replacement of remote calls with local tools is addressed in the next paragraph. The computation of maximal external sub-workflows is a performance optimization step discussed later in this paper.)

· *Transparent use of local and remote resources: *For most programs, *Tavaxy *allows its user to choose whether a task should run on local or remote computational resources. Furthermore, if a Taverna workflow is imported (e.g., from my Experiment), *Tavaxy *offers users an option to replace calls to remote web services automatically with calls to corresponding tools that run on a local computing infrastructure, or vice versa. (Note that almost all the workflows published on myExperiment are based on using remote services). Changing the default mode of invocation in either Taverna or Galaxy requires programming knowledge, and it is difficult to achieve by the non-programming scientist.

· *Simplified control and data constructs: Tavaxy *supports a set of advanced control constructs (e.g., *conditionals *and *iterations*) and data constructs (e.g., nested lists) and allows their execution on the local or remote computational infrastructures. The use of these constructs, which are referred to as “patterns” in *Tavaxy*, facilitates the design of workflows and enables further parallelization, where the data items passed to a node can be processed in parallel. The user of *Tavaxy *has the extra advantages of 1) adding these constructs to imported Galaxy workflows, and 2) using these constructs on the local infrastructures; features that are available only in Taverna and only for remote tools.

Beyond these integration issues, *Tavaxy *provides the following additional features that facilitate authoring and execution of workflows:

· *Enhanced usability: Tavaxy *uses flowchart-like elements to represent control and data constructs. The workflow nodes are annotated with icons to reflect if they are executed locally or remotely. The tool parameters can be defined either at the design- or run-time of the workflow. The data patterns offered in *Tavaxy *further facilitate the composition of workflows, making them more compact, and enable exploitation of local high performance computing infrastructure without any additional effort. Furthermore, each user has an account associated with its data and each workflow is further associated with its history as well as previously used datasets within the user account.

· *Modularity: Tavaxy *is modular; it separates the workflow composition and management modules from the workflow engine. Its workflow engine is a standalone application accepting both workflow definitions and data as input. This feature, as will be made clear later in the manuscript, is of crucial importance for implementing control constructs and for supporting cloud computing.

· *High performance computing infrastructure support: Tavaxy *can readily run on a computer cluster, once a job scheduler system (like PBS Torque or SGE) and a distributed file system (like NFS) are installed. The execution of parallel tasks is handled automatically by the workflow engine, hiding all invocation details.

· *Cloud computing support: Tavaxy *is cloud computing friendly, enabling users to scale-up their computational infrastructure on a pay-as-you go basis, with reduced configuration efforts. Through a simple interface within the *Tavaxy *environment, a user who has a cloud computing account (e.g., at the Amazon AWS platform) can easily instantiate the whole system on the cloud, or alternatively use a mixed mode where his local version of the system can delegate the execution of a sub-workflow or a single task to a *Tavaxy *cloud instance.

In the remaining part of this section, we will review basic concepts of workflow interoperability and *workflow patterns *that contributed to the design and development of *Tavaxy*.

### Related technical work

#### Workflow interoperability

Our approach described in this paper goes beyond the run-time “black-box” invocation of one system from the other, which was used in the work of [25,26] to enable interoperability between Galaxy and Taverna. To highlight the difference, the Workflow Management Coalition, WfMC, [27] defines eight models, or approaches, for achieving interoperability between workflow systems. These models can be grouped broadly into two major categories: 1) Run-time interoperability, where one system invokes the other system through APIs. 2) Design-time interoperability, where the two systems are based on a) the same model of computation (MoC); or b) the same languages (or at least translation between languages is feasible), or c) the same execution environment (or at least the existence of an abstract third-party middleware). These three design-time issues are discussed in detail in the paper of Elmroth et al. [28].

As discussed earlier, both Taverna and Galaxy have different models of computation and different languages. In this paper, we use ideas from the workflow interoperability literature and introduce the concept of patterns to integrate and execute Taverna and Galaxy workflows in *Tavaxy *at both run-time and design-time levels..

#### Workflow patterns

Workflow patterns are a set of constructs that model a (usually recurrent) requirement (sub-process); the description of these constructs is an integral part of the pattern definition. Workflow patterns, despite being less formal than workflow languages, have become increasingly popular due to their practical relevance in comparing and understanding the features of different workflow languages. As originally introduced in [29], workflow patterns were used to characterize business workflows and were categorized into four types: *control flowdata flowresource and operational*, and *exception handling *patterns. We note that the concept of patterns is in general applicable to scientific workflows. In [25], we used this concept for the first time to demonstrate the feasibility of achieving interoperability between Taverna and Galaxy. Our work in this paper extends this demonstrative work by providing a larger set of the patterns, and also by providing a complete implementation of them within a functional and usable system.

## Implementation

### Tavaxy model of computation and language

*Tavaxy *workflows are directed acyclic graphs (DAGs), where nodes represent computational tools and edges correspond to data flows or dependencies between them. The workflow patterns defined and used in *Tavaxy *have special meanings in this DAG, as will be explained in detail later in the pattern implementation subsection. The *Tavaxy *engine is based on a data flow model of execution [22,28,30-34], in which each node (task) can start computation only once all its input data are available. The *Tavaxy *workflow engine is responsible for keeping track of the status of each node and for invoking its execution when the predecessor nodes have finished execution and when its input data is completely available. When executing on a single processor, where tasks are executed sequentially, the order of task invocation can be determined in advance. This is achieved by traversing the DAG and scheduling a node (i.e., adding it to the ready queue) only if all the its predecessor nodes are already scheduled. As such, this scheduling is referred to as a *static *scheduling [34]. When executing on multiple processors, independent ready tasks can be executed in parallel. In this case, the *Tavaxy *engine keeps looking for ready tasks and launches them concurrently. On multi-core machines, the engine uses multi-threading to handle the execution of concurrent tasks. On a computer cluster, it passes the concurrent tasks to a job-scheduler, which in turn distributes them for execution on different cluster nodes. The default job-scheduler used in *Tavaxy *is PBS Torque, and it is set-up over a shared file system (NFS for local setting and S3 for cloud infrastructure) to guarantee availability of data for all cluster nodes.

A *Tavaxy *workflow is defined and stored in tSCUFL format, which is similar in flavor to the Taverna SCUFL format. However, there are two main differences between the two formats: 1) A node’s parameters are represented in tSCUFL by default as attributes of the respective tool, whereas they are considered as input items in SCUFL. 2) The workflow patterns (e.g., conditionals and iteration) are explicitly specified in tSCUFL but implicitly defined in SCUFL.

### Integrating Galaxy and Taverna workflows in Tavaxy

*Tavaxy *provides an easy-to-use environment allowing the execution of *Tavaxy *workflows that integrate Taverna and Galaxy workflows as sub-workflows. Such integration can be achieved at both *design-time *and *run-time*:

For run-time integration, *Tavaxy *can execute both Galaxy and Taverna (sub-) workflows ‘as is’, with no modification. For Galaxy workflows, this is straightforward, because the *Tavaxy *engine is compatible with the Galaxy engine and follows the same model of computation. For Taverna workflows, *Tavaxy *can execute a Taverna (sub-) workflow by invoking the Taverna engine through a command line interface that takes both the Taverna (sub-) workflow file and its data as input. The *Tavaxy *mapper component assures the correct data transfer between the Taverna engine and other nodes. This is achieved by setting source and destination directories and input/output file names in appropriate manners.

For design-time integration, *Tavaxy *imports and manipulates workflows written in either Galaxy or Taverna formats. *Tavaxy *can import a Galaxy workflow file to its environment, allowing its modification and execution. The engineering work for this step includes translation of the JSON objects of the Galaxy workflow to the tSCUFL format of *Tavaxy*. For Taverna workflows, the implementation addresses the differences in the model of computation and workflow languages. Specifically, the workflow engine of *Tavaxy *is a data-flow oriented one, with no *explicit *specification of control constructs, while the Taverna engine supports both data- and control-flow constructs.

The Taverna workflow language is SCUFL/t2flow but that of *Tavaxy *is tSCUFL. To overcome these differences, we use the concept of *workflow patterns *to 1) execute (“simulate”) the execution of Taverna control and data constructs on the data-driven workflow engine of *Tavaxy*; and 2) to provide a pragmatic solution to language translation where a Taverna (sub-) workflow is decomposed into a set of patterns that are then re-written in *Tavaxy *format. The following section introduces the *Tavaxy *workflow patterns and their implementation.

### Workflow patterns: Definitions and implementation

We divide the *Tavaxy *workflow patterns into two groups: control patterns and data patterns. In the remainder of this subsection, we define these patterns and their implementation on the *Tavaxy *data-flow engine.

#### Control patterns

**Figure 2:**
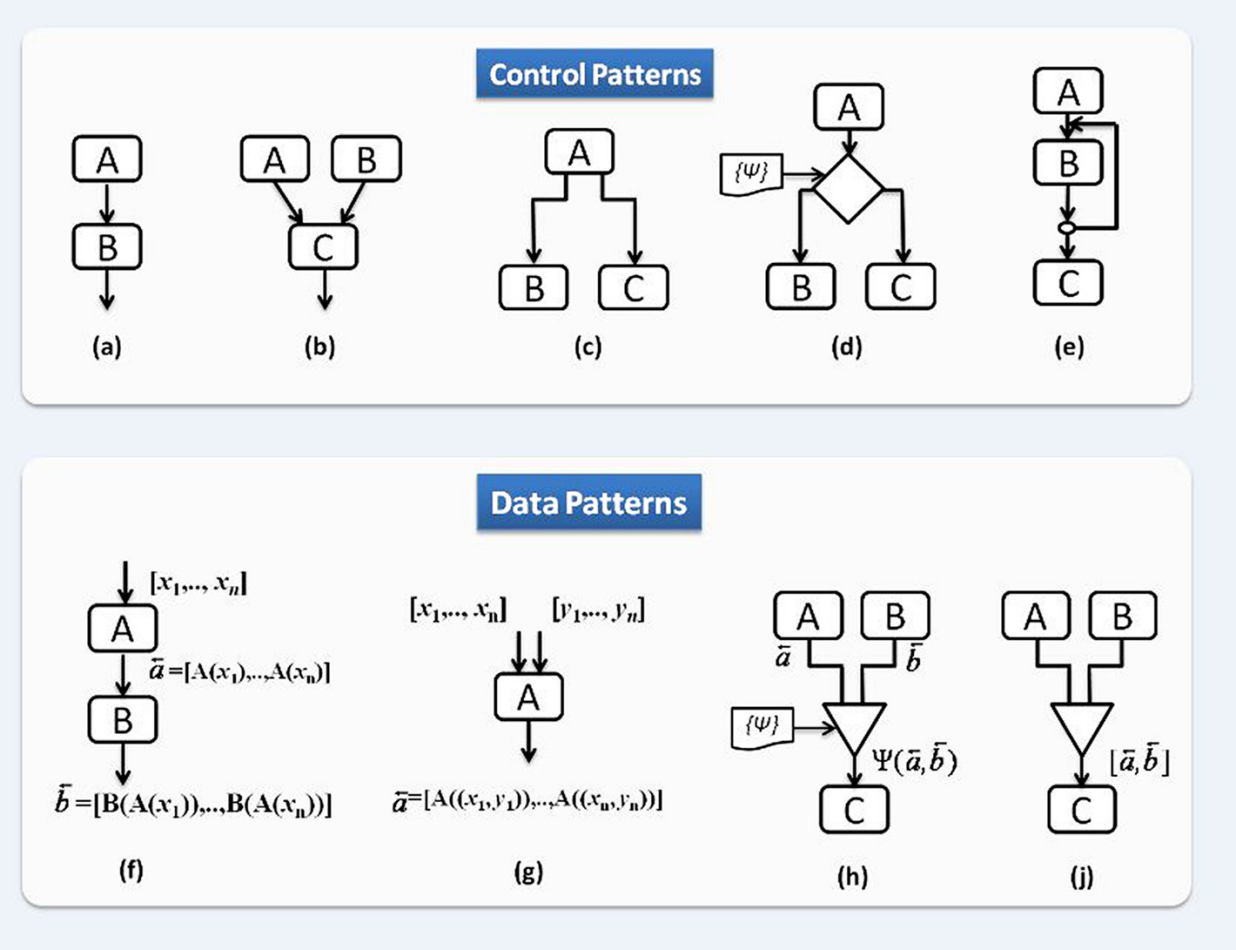
**Workflow patterns of Tavaxy.** Workflow patterns modeling the execution of workflow tasks. The parts (**a**), (**b**), (**c**), (**d**), and (**e**) represent the sequence (pipeline) pattern, the synchronous merge, the synchronous fork, multi-choice fork, and iteration control patterns, respectively. The part (**f**) shows how a list of data items is processed, and (**g**) shows dot/cross product operation. The parts (**h**) and (**j**) represent the data select and data merge patterns, respectively.

Control patterns specify execution dependencies between tasks. For most control patterns, data flow is still required and is defined as part of the control pattern specification itself. The following are the key control patterns used in *Tavaxy*:

1. *Sequence: *In this pattern, task *B *runs after the termination of task *A*, as shown in Figure 2(a). The data produced by *A *is subsequently processed by *B *and moves over an edge whose start is an output port at *A *and whose destination is an input port at *B*. The concept of ports makes it possible to select which pieces of data produced by *A *are passed to *B*. Desired execution dependencies involving no data can be achieved on the *Tavaxy *data flow engine by a special token (dummy output) from *A *to *B*. The current engine of *Tavaxy *does not support streaming, and the tasks are stateless, according to the discussion of Ludäscher et al. [31].

2. *Synchronous Merge: *A task is invoked only if all its predecessor tasks are executed; Figure 2(b) depicts this pattern with three tasks *A*, *B*, and *C*, where task *A *and *B *should be completed before *C*. This pattern also specifies that task *C *takes two inputs (one from *A *and another from *B*) and the data flowing from *A *and *B *to *C *goes to different input ports.

3. *(Parallel) Synchronous fork: *Figure 2(c) depicts this pattern with three tasks *A*, *B*, and *C*. Tasks *B *and *C *run after the execution of *A*. The data output from *A *flows according to one of two schemes, as specified by the user through settings of ports: 1) One copy of the data output of *A *is passed to *B *and another one to *C*. 2) Different data output items of *A *are passed to *B *and *C*. The tasks *B *and *C *can always run in parallel, because their input set is already available and they are independent.

4. *Multi-choice fork: *This pattern includes the use of an *if-else *construct to execute a task if a condition is satisfied. This condition is defined by the user through an implementation of a Ψ function. Figure 2(d) shows an example, where either *B *or *C *is executed, depending on the Ψ function, whose domain may include the input data coming from *A*. Note that the input data to *B *and *C*, which can come from any other node including *A*, is not specified in the Figure. Because this pattern specifies run-time execution dependencies, it is not directly defined over a data-flow engine. Therefore, we implemented this pattern on the *Tavaxy *engine by creating a special node containing a program that implements the switch function. The engine executes this node as a usual task. The program for switch pattern takes the following as input: 1) the multi-choice condition, and 2) the data to be passed to the next tasks. It then checks the condition and passes a success signal to the branch satisfying the condition and passes fail signal to the branch violating that condition. The success and fail signals are special tokens recognized by *Tavaxy *nodes.

5. *Iteration: *This pattern specifies repetition of a workflow task. In Figure 2(e), the execution of node *B*, which could be a sub-workflow, is repeated many times. The number of iterations can be either fixed or dependent on the data produced at each step. In each iteration, an output of task *B *can replace the corresponding input. For example, a parameter file can be passed to *B *and at each iteration this parameter file is modified and passed again to *B*. Node *C*, which represents any node that uses the output of *B*, is invoked only after the iteration pattern terminates. The iteration pattern is represented by a special node in *Tavaxy *and the associated program that implements it takes the following items as input: 1) the task (or sub-workflow) that iterates, 2) its parameters, 3) the termination criteria (defined by python script), and 4) information about feedback data. The iteration is implemented as a *do-while *loop, where the tasks in the body of the loop are encapsulated as a sub-workflow. *Tavaxy *is invoked recursively to execute this sub-workflow in each iteration. The output of the iteration pattern is specified by the user and is passed to the next task upon termination. The loop iterations are in general stateless; but the user can modify the included sub-workflow to keep state information.

#### Advanced data patterns and types

1. *(Nested) Lists: *In this pattern, the input to a node is a list of *n *items. The program associated with the node is invoked independently *n *times on each of the list items. Figure 2(f) shows an example where a list (*x*_*1*_, ..., *x*_*n*_) is passed to *A*. The output is also a list (*A(x*_*1*_*), ..., A(x*_*n*_*)*). Note that if the list option is not specified in the node, then the respective program is invoked once and the input list is handled as a single object, as in the sequence pattern. For example, a program for Primer design would consider a multi FASTA file as a list and is invoked multiple times on each item (sequence), while an alignment program would consider the sequences of the multi-FASTA file as a single object to build a multiple sequence alignment. In *Tavaxy*, it is possible to process the list items in parallel, without extra programming effort. Furthermore, a list can be a list of lists defined in a recursive manner, so as to support a nested collection of items, according to the notion of [9,32]. The jobs corresponding to the processing of every list item are stateless, according to the discussion of Ludäscher et al. [31]. However, the script implementing the list keeps track of the running jobs, and reports an error message if any job failed.

Over this list data type, we define a set of operators that can be used by the main program associated with the node.

· *Dot product: *Given two lists *A[a*_*1*_, ..., *a*_*n*_*] *and *B[b*_*1*_*,..,b*_*m*_], *n ≤ m *as input, a dot product operation produces the *n *tuples [*(a*_*1*_*,b*_*1*_*),..,(a*_*n*_*,b*_*n*_*)*] which are processed independently by the respective program, see Figure 2(g). (lists [*b*_*n+1*_, ..., *b*_*m*_] items are ignored.) This operation can be extended to multiple lists.

· *Cross product: *Given two lists *A[a*_*1*_, ..., *a*_*n*_*] *and *B*[*b*_*1*_, .., *b*_*m*_], *n < m *as input, a cross product operation produces the set of (*n × m*) tuples $\left\{(a_{i},b_{j})|,i\in [\text{1..}n],j\in [\text{1..}m]\right\}$, which are processed independently by the respective program. This option can be used, for example, for comparing two protein sets (each coming from one species) to each other to identify orthologs. If *A = B*, then we compare the set of proteins to themselves to identify paralogs.

The list operations are implemented by a generic tool-wrapper of *Tavaxy*. As we will explain later in the sub-section describing the architecture of *Tavaxy*, this wrapper is what is invoked by the workflow engine, and it is the one that invokes the program to be executed. The wrapper pre-processes the input and can make parallel invocations on different list items if *Tavaxy *is executing on a multiprocessor machine. The data collect pattern (specified below) can then be used to combine the results back in list format.

2. *Data select: *Consider Figure 2(h) with the three tasks *A*, *B*, and *C*. The data select pattern takes as input 1) Output data from *A *and *B*, denoted by $\vec{a}$ and $\vec{b}$, respectively. It takes also an implementation of a function Ψ that operates on properties of $\vec{a}$ or $\vec{b}$. Without loss of generality, the output of this pattern is $\vec{a}$, if $\Psi ((\vec{a},\vec{b}))$ is true, otherwise it is $\vec{b}$. The output of the pattern can be passed to another node *C*. This pattern is implemented in a similar way to the multi-choice pattern, where it specifies selection of certain data flow.

3. *Data collect (Merge): *This pattern, which is depicted in Figure 2(j), specifies that the data outputs of *A *and *B *are collected (concatenated) together in a list; i.e., the output is $\left[\vec{a},\vec{b}\right]$. Note that $\vec{a}$ or $\vec{b}$ could be a list of objects as well, which leads to creation of nested collections. This pattern is implemented in a similar way to the data select pattern, where data items are collected.

### Tavaxy architecture

**Figure 3:**
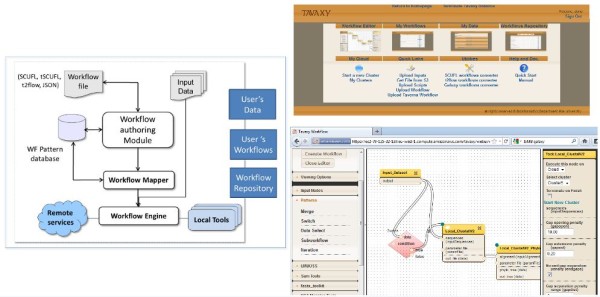
**Tavaxy architecture and interface.** Left: Tavaxy Architecture. The authoring module (workflow editor) is where users compose, open, and import workflows into Tavaxy. The imported workflows can be in tSCUFL, SCUFL, t2flow, JSON formats. The mapping module produces tSCUFL files to be executed by the engine. The engine invokes either local tools or remote services. Upper right: The main interface of the Tavaxy system containing links to the authoring module, user’s workflow, user’s data, workflow repository, and other utilities and cloud tools. Lower right: The workflow authoring module, where the switch pattern is depicted. The cloud symbol and the parameter port appear on the tool node. On the righthand panel, the user can choose if a tool runs locally or on the cloud.

Figure 3 (left) shows the architecture of *Tavaxy*, which is composed of four main components: 1) workflow authoring module, 2) workflow pattern database, 3) workflow mapper, and 4) workflow engine. On top of these components, we developed user accounts to maintain users workflows and data. We also developed a repository of public workflows that is shared between users. Figure 3 (upper right) shows the main *Tavaxy *page containing links to different system parts and utilities.

#### Workflow authoring tool and language

The *Tavaxy *workflow authoring module (workflow editor) is a web-based drag-and-drop editor that builds on the look and feel of Galaxy with two key modifications. First, it supports a user-defined set of workflow patterns that are similar to those used in a traditional flowchart. Second, it allows users to tag which workflow nodes execute on the local infrastructure and which execute using remote resources. For each node, there is a form that can be used to set the node’s parameters. Furthermore, each node has a specific port that can accept a parameters file that can be used to over-write parameter values set through the web-interface. The use of a parameters file allows changing of the value of parameters at run time. Figure 3 (lower right) shows the *Tavaxy *authoring module and highlights some of its key features.

#### Workflow mapper

The workflow mapper performs the following set of tasks:

· The mapper parses the input tSCUFL file and checks its syntax. It translates the Galaxy JSON format and TavernaSCUFL format to the *Tavaxy*tSCUFL format. Depending on user choices, it can replace remote Taverna calls with calls to corresponding local tools. The nodes that are still executed remotely by the Taverna engine will be encapsulated as a sub-workflow. Each sub-workflow is then associated with a *Tavaxy *node that invokes the Taverna engine so as to execute the corresponding sub-workflow. The mapper sets the names of the sub-workflow input and output files in an appropriate manner so that the data correctly flows between the nodes. Additional file 1 (in the supplementary material) contains the re-writing rules for translating SCUFL to tSCUFL formats, including control constructs and replacement of remote services with local tools.

· The mapper optimizes the execution of a workflow by identifying the tasks that will be executed by the Taverna engine and aggregating them into *maximal external sub-workflows.*. A sub-workflow is called *external *if it includes only Taverna nodes and it is *maximal *if no extra external nodes can be added to it. The mapper determines the maximal external sub-workflows using a simple graph-growing algorithm, where we start with a sub-graph composed of a single Taverna node and keep adding external nodes to this sub-graph provided that there are edges connecting the new nodes to the sub-graph and no cycles are introduced. To find the next maximal external sub-workflow, we move to the next non-processed external node. After sub-workflow identification, the mapper encapsulates each maximal external sub-workflow in a new node and adjusts the input and output ports in an appropriate manner. Accordingly, the Taverna engine is invoked only once for each maximal external sub-workflow, which avoids the overhead of multiple Taverna calls. Note that Taverna uses multi-threading to handle execution of independent tasks, including remote invocations. Hence, the use of maximal external sub-workflows with remote calls entails no loss in efficiency.

#### Workflow engine

The *Tavaxy *engine is based on the data flow model of execution discussed earlier in this section. It is written in Python, based on some Galaxy functions to save development time. The *Tavaxy *engine (compared to the Galaxy engine) is standalone and not tightly coupled with the web-interface and database-interface; i.e., it can be invoked programatically or using a command line interface. Furthermore, it can invoke itself in a recursive manner, which enables the implementation of different patterns and integration of heterogeneous workflows. By building on some of core features of Galaxy engine, the *Tavaxy *engine can be regarded as an extended and engineered version of that of Galaxy. The Taverna engine is invoked as any program (secondary engine) to achieve run time interoperability with Taverna workflows and to use it in invocation of remote services.

All local tools in *Tavaxy *are wrapped within a generic wrapper that is invoked by the engine.

This wrapper is responsible for the following:

· The wrapper decides whether the associated tool is executed or not, depending on reception of a special token (dummy data). The special token can correspond either to 1) execution dependency or 2) “do-not-execute” or “fail” signal from the preceding node, as in the case of the multi-choice pattern. In the former case, the wrapper executes the respective computational tool, while in the latter case, it will not invoke the tool and further passes the token to the output ports.

· It handles the list patterns by determining the list items, executing list operations, and invoking the respective program (in parallel) on list items.

· It uses cloud computing APIs to execute tasks on cloud computing platforms. The use of cloud computing is discussed below in more detail.

#### Workflow pattern database

The workflow pattern database stores the definition and implementation of the workflow patterns used in *Tavaxy*. It also stores how the nodes associated with these patterns are rendered on the workflow authoring client. This pattern database is extensible by the user, who can define new patterns according to the rules of the *Tavaxy *system.

### Use of cloud computing

**Figure 4:**
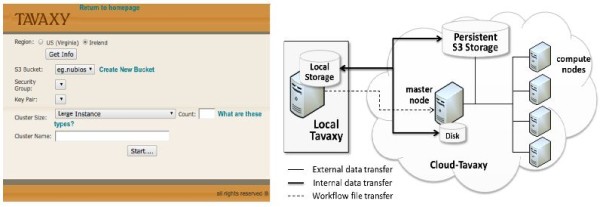
**Use of cloud computing in Tavaxy.** Left: The web interface for setting the computer cluster on the cloud. Right: The architecture of Tavaxy showing the local and cloud versions of the system. The data flows from the local version to either the mounted disk attached to the main machine or to the persistent S3 storage. The S3 storage serves two purposes: 1) persistent storage and 2) shared storage for the computer cluster.

As briefly mentioned before in the introduction, we provide three modes for using cloud computing: 1) whole system instantiation, 2) sub-workflow instantiation, and 3) tool (service) instantiation. To further simplify the use of the first mode, we installed an instance of *Tavaxy *(including the whole web-interface and tools) on an Amazon AWS virtual machine and deposited a public image of it at the Amazon web-site. A user who has an Amazon account can directly start the image and use it. Based on Amazon APIs, this image can establish a computer cluster upon its activation. The user can specifically define the type of nodes (e.g., large or extra large) and their number. The Amazon S3 storage is used as a shared storage for the computer cluster. We developed several interface functions that manage data transfer among the compute nodes and the shared storage of the cluster at run time. Figure 4(left) shows a screen shot of the *Tavaxy *interface page, where the user can configure the cluster and storage.

In the second mode, the user already has a *Tavaxy *version installed on his local machines (called *local Tavaxy*) and delegates the execution of one or multiple sub-workflows to be executed on the cloud. To support this scenario, a lightweight version of *Tavaxy *has been deposited at the Amazon platform as a virtual machine image. From a simple user interface in the local *Tavaxy*, the user can start and configure a cloud cluster using the prepared *Tavaxy *image.

At run-time, the local version of *Tavaxy *communicates with the cloud counterpart, using a simple asynchronous protocol (similar to the REST protocol), to send the sub-workflow, execute it, and retrieve the results. The input and output data related to such a sub-workflow flow according to one of two scenarios:

1. The input data is sent to the mounted disk of the main cloud machine along with the workflow to be executed. After processing, the output is sent back to the local *Tavaxy*. After termination of the machine, the input and result data are lost, unless they are moved by the user to a persistent storage. This scenario is useful in case no computer cluster is needed.

2. The input data is sent to a shared volume in the persistent S3 storage (this can be done offline), where the compute nodes of the computer cluster can easily access it. Because reads and writes to S3 require the use of Amazon APIs, we developed special scripts to facilitate this access between the local *Tavaxy *and S3 on one side and between the compute nodes and S3 on the other side. After execution of the sub-workflow, a copy of the output is maintained on the S3 and another copy is sent to the local *Tavaxy *to complete the execution of the main workflow.

The third mode is a special case of the second mode, where the user can delegate the execution of only a single task to the cloud. For this mode, we also use a simple protocol to manage the data transfer and remote execution of the task on the cloud. Figure 4(right) shows the architecture of the cloud version of *Tavaxy *and the data flows among its components.

## Results and discussion

### Accessing Tavaxy

There are different ways to access and use the *Tavaxy *system from its main home page:

1. Downloadable version: The whole *Tavaxy *system, with all features described in this manuscript, can be downloaded for local use. The bioinformatics packages are provided in a separate compressed folder, because we assume that some users already have installed the packages of interest on their local systems and just need the *Tavaxy *system. The packages currently include about 160 open source tools, coming from EMBOSS [35], SAMtools [36], fastx [37], NCBI BLAST Toolkit [38-40], and other individual sequence analysis programs. Addition of extra tools is explained in the *Tavaxy *manual.

2. Web-based access: We offer a traditional web-based interface to a *Tavaxy *instance for running small and moderate size jobs. For large scale jobs, we recommend the use of cloud version.

3. Cloud-computing based access: In this mode, each user creates a *Tavaxy *instance with the hardware configuration of choice on the AWS cloud. The interesting feature in this model is that multiple users may have multiple *Tavaxy *systems, each with different configuration (number and type of ‘virtual’ machines). The *Tavaxy *instances on the cloud already include the 160 tools currently tested. They also include a number of databases to be used with the cloud machines, such as the NCBI (nucleotide and protein) and swissprot databases.

### Pre-imported workflows

At the time of preparing this manuscript (June 2011), the Taverna repository myExperiment contained 557 workflows in SCUFL (Taverna1) format and 554 workflows in t2flow format (Taverna2). By manual inspection, we found that 296 workflows (96 in SCUFL format and 200 in t2flows format) are related to the sequence analysis domain, which is the main focus of this version of *Tavaxy*. To help the community, we already imported all these workflows into the *Tavaxy *environment, and arranged them in a special web-accessible repository for public use. We also provided the user with optimized versions of the sequence analysis workflows, where many of the web-services are replaced with local invocations of the corresponding local tools distributed with *Tavaxy*. We also imported all public Galaxy workflows from the Galaxy Public Pages and added them to this repository. The workflows imported from both the Taverna and Galaxy repositories are included in the *Tavaxy *system, and will be kept up-to-date on its web-site. These workflows can serve as “design patterns” that can can be used to speed up workflow development cycle, when developing more complex workflows.

### Experiments overview

In the following sub-sections, we introduce two case studies that demonstrate the key features of *Tavaxy*. In the first case study, we demonstrate 1) how Taverna, Galaxy, and *Tavaxy *sub-workflows can be integrated in a single *Tavaxy *workflow, highlighting both the integration capabilities and use of workflow patterns; and 2) the optimization steps included before the execution of imported workflows and their effects on the performance of the system. In the second case study, we demonstrate 1) the use of *Tavaxy *for a metagenomics workflow based on NGS data; 2) the advantages of using advanced data patterns in facilitating the workflow design and supporting parallel execution; 3) the speed-up achieved by using local HPC infrastructure; and finally 4) the efficient and cost-saving use of cloud computing.

### Case study I: Composing heterogeneous sub-workflows on Tavaxy

**Figure 5:**
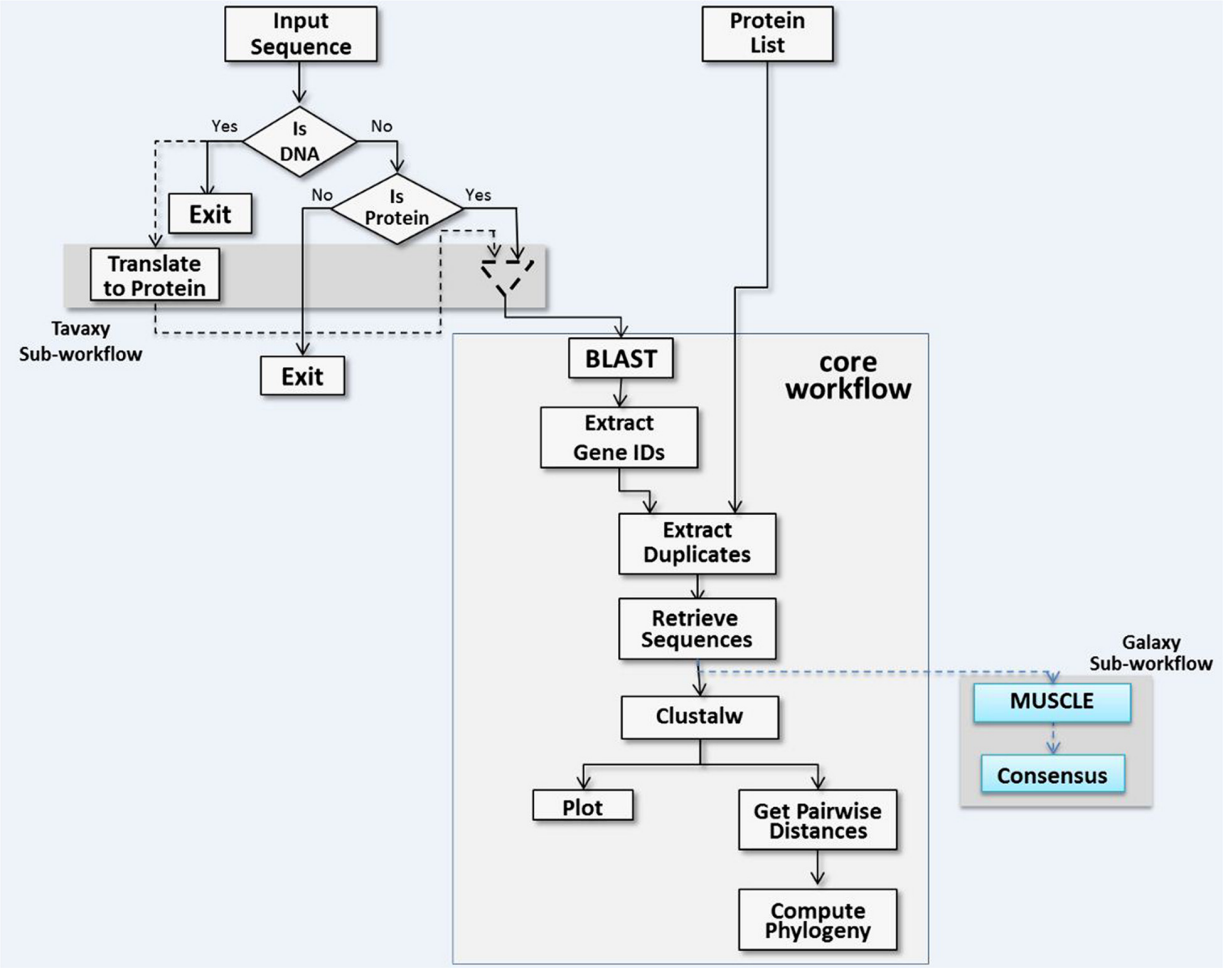
**Protein analysis workflow.** Workflow for finding and analyzing homologous protein sequences. The highlighted parts are extra sub-workflows from Galaxy and Tavaxy, and the remaining parts correspond to a Taverna workflow already deposited at myExperimentweb-site.

Figure 5 shows a workflow for finding homologous protein sequences and analyzing them. The workflow starts with reading a DNA/protein sequence from the user. If the input is a DNA sequence, it is translated to a protein sequence. The input sequence is passed to BLAST [38,39] to find similar sequences. The output of BLAST, which is a list of Genbank IDs, is then compared to a user-provided list of sequence IDs to exclude common sequences from the output list. The protein sequences of the exclusive IDs are then retrieved and passed to the programs ClustalW [41,42] and MUSCLE [43] for computing multiple alignment. ClustalW is also used for computing a phylogenetic tree.

**Figure 6:**
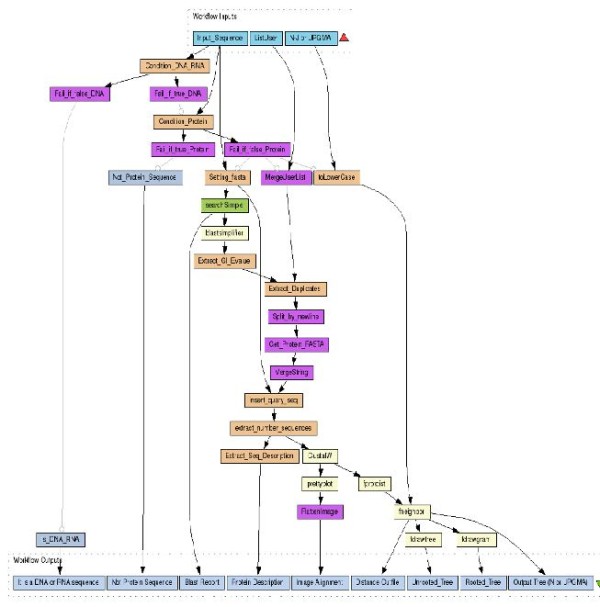
**Taverna implementation of the protein analysis workflow.** Taverna implementation of the workflow in Figure 5. All program parameters (e.g., BLAST tool to be used and UPGMA NJ option) are considered as input to the workflow. High resolution versions of the figures of this paper are available in Additional File 2.

Searching the myExperiment repository, there is already an existing Taverna implementation for most of the desired workflow, deposited under the name “workflow_for_protein_sequence_analysis” [44], and Figure 6 shows its implementation as it appears in the Taverna authoring module. The missing functionality in this Taverna workflow are the two parts highlighted in Figure 5, including the parts for translating the DNA sequence into protein sequence and the one for MUSCLE-consensus. In the original Taverna implementation, the software tools BLAST, ClustalW, and phylogeny plotting are invoked through web-service interfaces. The other intermediate steps are executed by built-in Taverna programs.

**Figure 7:**
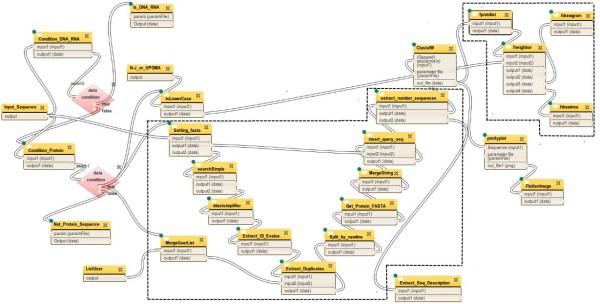
**Imported Tavernaworkflow in Tavaxy.** The imported Tavernaworkflow in Figure 6. The Tavaxy switch pattern is explicitly represented. The switch patterns are represented by diamond shapes. The upper switch pattern checks if the input sequence is DNA. If false, the lower switch pattern checks if it is a protein one. The dashed polygons mark two maximal external sub-workflows which will be encapsulated in the optimization step, as in Figure 8.

**Figure 8:**
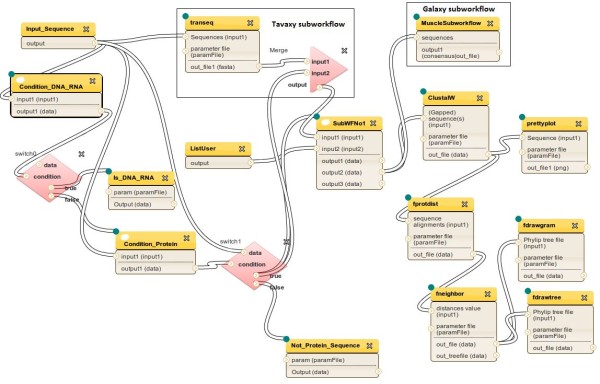
**Hybrid and optimized workflow in Tavaxy.** The workflow in Figure 7 after optimization and augmentation with extra components. Sub-workflows 1 and 2 are the maximal external sub-workflows marked in Figure 7 by dashed polygons. The extra Galaxyworkflow and Tavaxy nodes are also shown.

We downloaded the Taverna workflow and imported it into *Tavaxy*; Figure 7 shows the same workflow in the *Tavaxy *environment. At this step, the user may choose to execute this workflow as it is from *Tavaxy*, or may choose to optimize the execution of the workflow and/or customize it by adding further tasks. For example, for this workflow, the user can replace web-services with equivalent locally installed tools through a simple user interface. The workflow mapper carries out this replacement and can, according to user choices, coalesce the remaining Taverna tasks into maximal sub-workflows, as described earlier in the *Tavaxy *implementation section. In this example, we decided that the ClustalW and the phylogeny analysis parts of the workflow run on the local infrastructure, while the BLAST part still runs remotely. Figure 8 shows the optimized version of this workflow, where the maximal Taverna sub-workflows are computed. The functionality of this workflow can be augmented with further tasks. First, we re-used a native Galaxy (sub-) workflow that computes multiple alignment using the MUSCLE program and computes the consensus sequence. Second, we added a *Tavaxy *sub-workflow, in which the DNA sequences are translated into protein sequences, instead of ignoring processing them. To link the translated sequences to the other parts of the workflow for further analysis, the *data merge *pattern is used to pass the protein sequences. These extra parts are highlighted in Figures 5 and 8.

#### Measuring the performance

We conducted an experiment to evaluate the overhead associated with invoking the Tavern engine to execute remote tasks, before and after the optimization step. We used the original Taverna workflow and its imported version (i.e., we did not use the extra Galaxy and Tavaxy sub-workflows shown in Figure 8), with the list of input protein IDs (for checking duplicates) being empty. We measured the running time of this workflow with respect to three different execution scenarios. In the first scenario, the original Taverna workflow was executed on Taverna, where the tasks are executed remotely. In the second scenario, the workflow was executed after replacing the remote tools with equivalent local ones (except for BLAST). In the third scenario, the workflow was executed after conducting the optimization step to reduce the number of invocations of the local Taverna engine.

For this experiment, we used the example protein sequence distributed with the Taverna workflow on myExperiment. We also used another set of proteins used by Kerk et al. [45] to update the Protein Phosphatase database with novel members. The basic idea of their work is to use a set of representative human proteins from different phosphatase classes to identify homologs from different genomes. It is worth mentioning that the workflow at hand automates most of the manual steps conducted in the study of Kerk et al. [45]. Hence, it can be used to systematically and automatically revisit the protein phosphatase repertoire.

Table 1 shows the average running times for the different execution scenarios specified above. The experiments were conducted on an 8 core machine (AMD Opteron 1.2 GHz processors) and 64 GB RAM. It can be noted that the workflow is not compute-intensive, as it handles one protein sequence at a time and the amount of transferred data on the web is not too large. Therefore, it does not take much time to execute on Taverna. Running the workflow from *Tavaxy *after using local tools without optimization led to higher execution time due to the overhead associated with the invocation of the Taverna engine at each step. After optimization into maximal external sub-workflows, this time decreased and the overhead was minimized. We note that the time on *Tavaxy *for the last five proteins is slower than that of Taverna. The reason for this is that these proteins are shorter than the others, which means short running time. Hence, the overheads of calling Taverna outweigh the gain in saving data transfer and using local tools. It is important to note that this overhead is proportional to the complexity of the workflow and not to the data size. This means that it would be neglected for time consuming experiments.

**Table 1:** The average running times for the protein workflow

**Protein Name**	**Taverna**	**Tavaxy-local**	**Tavaxy-optimized**
NP_061857.3 (gi|239047414)	4:10	8:05	3:04
NP_203747.2 (gi|37674210)	4:17	8:45	3:16
NP_060327.2 (gi|24586675)	4:36	7:36	3:21
Q9UNH5.1 (gi|55976620)	4:31	7:24	3:13
O60729.1 (gi|55976216)	4:35	7:18	3:03
P30304.2 (gi|50403734)	2:50	8:30	3:01
P30305.2 (gi|21264471)	2:50	8:30	3:01
NP_001781.2 (gi|125625350)	1:48	7:22	3:20
NP_054907.1 (gi|7661832)	2:08	7:36	2:54
Example seq.	1:21	7:17	2:44

The average running times in minutes for different protein sequences and for different execution scenarios of the protein homology workflow. The last protein, Example seq., is the example protein distributed with the Tavernaworkflow. The other proteins are from the study of [45].

Despite the differences in the design of the Taverna, Galaxy, and *Tavaxy *engines, we performed an extra experiment to compare their performance. We used the sub-workflow in this case study, including the BLAST and ClustalW calls, as a test workflow. This sub-workflow is highlighted in Figure 5 and denoted as ‘core workflow’. For Taverna, we used the local installation of the programs and we wrote special shell scripts to run them on the local infrastructure. (This is not a usual use case for using Taverna and it is not a straightforward task for the non-programming scientist.) The results of this experiment, which are shown in Table 2, indicates that the performance of the three systems is very similar. We note a little overhead when using Galaxy and *Tavaxy*, because the engines of both systems are designed for multiple users, while the Taverna engine is desktop based serving a single user. We also note that the *Tavaxy *engine, as expected, is a little slower than that of Galaxy. This can be attributed to the overhead associated with the extra wrapper module developed for handling the patterns and cloud functionalities. Note that these overheads are proportional to the workflow size, and would be negligible for large datasets.

**Table 2:** The average running times for protein homology sub-workflow on the Taverna, Galaxy, and Tavaxy systems

**Database**	**Sequence**	**Taverna (local)**	**Galaxy**	**Tavaxy**
0swissprot	NP_061857.3	0:32	0:38	0:37
	NP_203747.2	0:31	0:32	0:34
	NP_060327.2	0:33	0:40	0:41
	Q9UNH5.1	0:21	0:23	0:23
	O60729.1	0:17	0:23	0:23
	P30304.2	0:20	0:15	0:25
	P30305.2	0:20	0:25	0:28
	NP_001781.2	0:19	0:25	0:26
	NP_054907.1	0:18	0:22	0:26
	Example Seq.	0:15	0:17	0:19
refseq	NP_061857.3	2:56	3:20	3:15
	NP_203747.2	2:58	3:17	3:28
	NP_060327.2	2:12	2:00	2:02
	Q9UNH5.1	1:59	2:01	2:05
	O60729.1	1:50	1:39	1:42
	P30304.2	2:51	2:53	2:56
	P30305.2	2:12	2:21	2:22
	NP_001781.2	1:50	1:57	1:59
	NP_054907.1	1:53	2:01	2:04
	Example Seq.	1:46	1:43	1:47

The average running times (in minutes) of the workflow involving BLAST and ClustalWfor the protein sequences in Table 1. The whole workflow runs on local infrastructure. The queries are performed against the swissprot and NCBI refseq databases.

### Case study 2: A metagenomics workflow

**Figure 9:**
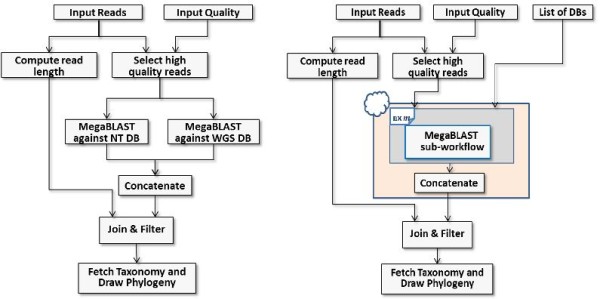
**Metagenomics workflow.** Left: Metagenomics workflow as originally provided by Galaxy. Right: the re-designed version of this workflow using the list pattern of Tavaxy.

Figure 9 (left) shows a flow chart representation of a metagenomics workflow deposited on the Galaxy public pages [46,47]. The input to this workflow is a set of NGS reads and associated quality data. The workflow starts with quality check of the reads and computation of their lengths. The high quality reads are queried, using the MegaBLASTtool [40], against two different databases chosen by the user. The reads with good alignment coverage are retained for further analysis. Finally, the taxonomical information of the successful reads are extracted from the alignment file and a taxonomy tree is plotted.

In the original implementation of this workflow on Galaxy [46,47], and as depicted in the schematic representation of Figure 9, we can identify two issues: First, the input reads are passed to MegaBLAST is a single multi-FASTA file which implies sequential processing of the queries against the database. Second, there are two nodes for MegaBLAST: one to consider the NCBI_WGS database and the other to consider the NCBI_NT database. To query more databases in Galaxy, additional nodes should be manually added; this will yield a bulky workflow for a large number of databases. In *Tavaxy*, we can enhance the design and execution of this workflow with respect to these two issues.

For the first issue, we use the *Tavaxy list pattern *in association with MegaBLAST so that the input multi-FASTA file is handled as a list of items. This will immediately lead to parallelization of this step. A list item could be a single FASTA sequence or a block of multiple FASTA sequences. We recommend that the input reads are divided into a list of *n *blocks, each of size *k *sequences. The parameter *k *is set by the user and it should be proportional to the number of processors available. (The list is defined by a special node and its items (blocks) are separated by a special user-defined symbol.) When the workflow with the list pattern is executed, multiple versions of MegaBLAST will be invoked to handle these blocks in parallel.

For the second issue, concerning the simple integration of more databases, we will use only just one MegaBLAST node and create a list of input databases. This list is passed as input to the MegaBLAST node. To ensure that each read is queried against all given databases, we use the *cross product *operation defined over the list of databases and the list of input sequences. For *m *databases and *n *blocks, we have *n × m *invocations of MegaBLAST, which can be handled in parallel without extra coding effort.

**Figure 10:**
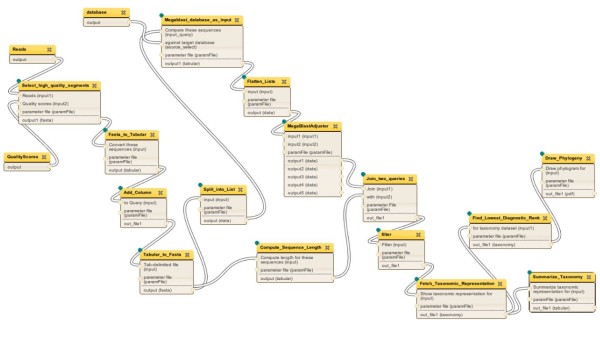
**The enhanced metagenomics workflow.** The enhanced metagenomics workflow as implemented in Tavaxy.

Figure 9 (right) shows a schematic representation of the enhanced workflow with the list pattern. Figure 10 shows the implementation of the enhanced workflow in *Tavaxy*. In this figure, the special node “split_into_list” defines the list items from the multi-FASTA file.

#### Measuring the performance

We tested the performance of the enhanced metagenomics workflow on a computer cluster using two datasets. The first was the dataset used by Huson et al. [48], constituting a metagenomic survey of the Sargasso Sea [49]. This dataset, which represents four environmental samples, is composed of 20,000 Sanger reads, where 10,000 come from Sample 1 and another 10,000 come from Samples 2–4. The second dataset is the windshield data set of [46], which is composed of two collections of 454 FLX reads. These reads came from the DNA of the organic matter on the windshield of a moving vehicle that visited two geographic locations (trips A and B). We used the reads of the left part of the windshield experienced both trips. The number of reads are 70343 (≈ 15.7 Mbp) and 89783 (18 Mbp) for trips A and B, respectively. For MegaBLAST, we used the NCBI_HTGS, NCBI_NT, and NCBI_ENV datasets.

Table 3 shows the average running times over a computer cluster of different compute nodes. (The cluster is composed of three machines, each with 8 cores (AMD Opteron 1.2 GHz processors), and 64 GB RAM, connected with a 1Gb Ethernet switch.) In this experiment, the list pattern divided the input data into 11 blocks, each with size ≈ 1000 sequences in case of the Sargasso data and ≈ 7000 sequences in case of the windshield data. (For a cross product with 3 databases, we have 33 jobs in total.) From the table, it can be seen that the running times decrease with the increased number of cores.

**Table 3:** The average running times of the metagenomics workflow on local infrastructure

**Dataset**	**Cores**					
	1	2	4	8	16	32
Windshield Trip A (left)	163	86	48	31	21	15
Windshield Trip B (left)	204	98	55	35	21	16
Sargasso Sea (Sample 1)	109	59	35	21	13	10
Sargasso Sea (Samples 2–4)	113	67	39	23	14	10

The average running times in minutes for varying numbers of processors (cluster cores) on the local infrastructure.

#### Use of cloud computing

We used the cloud computing features of *Tavaxy *on the sub-workflow level to execute the metagenomics workflow. The purpose is to test the use of cloud computing in terms of execution time and cost of computation. Here, we focused on the sub-workflow mode of using cloud computing, because it demonstrates the case. We decided to run the sub-workflow involving MegaBLAST with the list pattern on the cloud because it is the most compute-intensive part in this workflow. From the *Tavaxy *interface, we established a computer cluster on the AWS cloud. Each node includes a copy of the databases needed by MegaBLAST. The shared S3 cloud storage is attached to the cluster to maintain the output and intermediate results. For this experiment, we used Amazon instances of type “Extra Large”, with 8 cores (≈1.2 GHz Xeon Processor), 15 GB RAM, and 1,690 GB storage. The establishment of the cluster with the storage took a few minutes from the machine images.

Table 4 shows the execution times of the workflow for the same datasets mentioned before using different cluster sizes on the cloud. It also includes the monetary cost of running this workflow, for each cluster size. It is interesting to see that the use of more machines led to faster running time and reduced cost. In our case, the four machines (with total 32 cores) working in parallel run for less than one hour and cost totally $2.7. This is cheaper and faster than using a single machine that runs for about 6 hours and costs $4.1.

**Table 4:** The average running times of the metagenomics workflow on the AWS cloud

**Dataset**	**Cores**				
	1	4	8	16	32
Windshield Trip A (left)	330 ($4.1)	82 ($4.1)	34 ($4.1)	18 ($1.4)	14 ($2.7)
Windshield Trip B (left)	371 ($4.8)	91 ($4.8)	40 ($4.1)	21 ($1.4)	15 ($2.7)
Sargasso Sea (Sample 1)	252 ($3.4)	60 ($3.4)	22 ($3.4)	15 ($1.4)	9 ($2.7)
Sargasso Sea (Samples 2–4)	299 ($3.4)	73 ($3.4)	26 ($3.4)	16 ($1.4)	10 ($2.7)

The average running times in minutes for a computer cluster on the cloud. The number in bracket is the computation cost in US Dollars for the US-East site with $0.68 per hour (2011 AWS price list). (Note that partial computing hour of an instance is billed on Amazon as a full hour.)

## Conclusions

In this paper we introduced *Tavaxy*, a stand-alone pattern-based workflow system that can also integrate the use of Taverna and Galaxy workflows in a single environment, enabling their modification and execution. The *Tavaxy *integration approach is based on the use of hierarchical workflows and workflow patterns. *Tavaxy *also supports the use of local high-performance computing and the use of cloud computing. The focus of the current version of *Tavaxy*is on simplifying the development of sequence analysis applications, and we demonstrated its features and advantages using two sequence analysis case studies. Future versions of the system will support further applications in transcriptomics and proteomics.

We also introduced a set of advanced data patterns that simplify the composition of a variety of sequence analysis tasks and simplify the use of parallel computing resources for executing them. In future work, we will extend the available patterns to support more complex sequence analysis tasks, as well as other application domains. *Tavaxy*is currently shipped with its own repository of pre-imported Tavernaand Galaxy workflows to facilitate their immediate use. This repository can be regarded as a set of “design patterns” that can help in speeding up composition of more complex workflows.

In the current version of *Tavaxy*, we have set up the system for use on a traditional computer cluster on the AWS cloud. We have not yet investigated other HPC options, such as the Amazon Elastic MapReduce or the use of GPUs.

In future versions of *Tavaxy *we will investigate the use of these options to support efficient execution at the sub-workflow and task levels. We will also investigate the use of other cloud computing platforms.

Finally, we believe that one of the key advantages of *Tavaxy *is that it provides a solution that consolidates the use of remote web-services, cloud computing, and local computing infrastructures. In our model, the use of remote web-services is limited to only those shared tools that cannot be made locally available, the use of a local infrastructure supports the execution of affordable tasks, and the use of cloud computing provides a scalable solution to compute- and data-intensive tasks.

## **Availability and requirements**

**0.0.0.1. Project name:***Tavaxy*.

**0.0.0.2. Project home page:** http://www.tavaxy.org.

**0.0.0.3. Operating system(s):** Linux.

**0.0.0.4. Programming language:** Python, C, Java script, JSF

**0.0.0.5. Other requirements:** Compatible with the browsers FireFox, Chrome, Safari, and Opera. See the manual for more details.

**0.0.0.6. License:** Free for academics. Authorization license needed for commercial usage (Please contact the corresponding author for more details).

**0.0.0.7. Any restrictions to use by non-academics:** No restrictions.

## Competing interest

The authors declare no conflict of interest.

## Authors’ contributions

MA led the *Tavaxy *project. MA and MG contributed to theoretical developments of the architecture and workflow patterns which form the basis of *Tavaxy*. SA and MA developed and tested the software and implemented the workflows. All authors wrote and approved the manuscript.

## Supplementary Material

Additional file 1 **Re-writing rules for translating SCUFL to tSCUFL.** A PDF file describing the re-writing rules for translating a Tavernaworkflow in SCUFL format into Tavaxy workflow in tSCUFL format. Click here for file

Additional file 2 **Paper figures in original size.** Compressed folder containing the paper figures in original size for better visualization. Click here for file
