# Supplementary material for: Tavaxy: Integrating Taverna and Galaxy workflows with cloud computing support
Source: BMC Bioinformatics. 2012 May 4;13:77. doi: 10.1186/1471-2105-13-77 (PMC3583125; doi:10.1186/1471-2105-13-77)
Supplement: Additional file 1 — Re-writing rules for translating SCUFL to tSCUFL. A PDF file describing the re-writing rules for translating a Tavernaworkflow in SCUFL format into Tavaxy workflow in tSCUFL format. [file 1471-2105-13-77-S1.pdf]

Supplementary File 1

Translating Taverna Constructs into Tavaxy Constructs

Mohamed Abouelhoda Shadi Alaa Issa Moustafa Ghanem

1 Introduction

1.1 Taverna language

Workflows in Taverna are DAGs (Directed Acyclic Graphs), where the nodes are the tasks and edges represent data or control flow. A DAG corresponding to a workflow is represented either in the SCUFL (Simple Conceptual Unified Flow Language) or t2flow format. Because SCUFL and t2flow are very similar and follow the same principles, we will focus in this appendix on showing how to transform SCUFL format to Tavaxy format. In SCUFL, the graph nodes are referred to as *processors*, and edges between nodes as *links*. There are four types of processors in Taverna:

1. *Tool processor*: This processor invokes a tool or a web-service.

2. *Data source*: This node receives input data. A multi-input workflow contains multiple data sources.

3. *Data sink*: This node captures output data. A multi-output workflow contains multiple data sinks.

4. *String constant*: This processor includes a string written by the user in the design/coding time. This processor can be regarded as a program that outputs the included string to the succeeding nodes when the workflow runs.

Data source and string constants are also used in Taverna to pass parameter values to different tasks. (Note that the only way to pass parameters to a tool in Taverna is through data nodes.) The directed edges/arcs between the nodes that pass the output of one processor as input to another processor are called *data links*.

Taverna supports the following control flow constructs for organizing control flow operations:

- *Dependency (coordination) link*: It is an edge connecting two nodes with no data flowing on it in order to simply indicate sequential dependency between the two nodes.

- *Conditional branching*: It is an *if-else* construct, with two branching output channels, where a special 'success' token is passed to one branch and another 'failure' token is passed to the other. In SCUFL, the conditional construct is not introduced explicitly to the user. Rather, the user has to implement it using *node failure* and *dependency links* mechanisms, as will be explained later in this document.

- *Iteration:* Taverna supports iterative execution of a processor (processor could also include a sub-workflow). The user is not allowed to extend edges from any node to another previous one in an arbitrary manner, to avoid generation of cycles, and hence maintaining the DAG

structure. The iteration should encapsulate a processor with defined input and output. Like the conditional construct, the iteration construct is not introduced explicitly to the user; the user has to implement it using *retry*, *node failure* and *dependency links*, as will be explained later in this document.

More information about the SCUFL model and its implementation are available at `http://www.ebi.ac.uk/~tmo/mygrid/XScuflSpecification.html` and `http://www.taverna.org.uk/developers/taverna-1-7-x/architecture/scufl`

A simple SCUFL example:

Consider the following workflow that receives a DNA sequence in any standard format and performs the following two steps: 1) the program seqret, which is part of the EMBOSS package, is used to read a DNA sequence from the user and transforms it to FASTA format. 2) the output of seqret is passed to the program BLAST as a query. Both the programs seqret and BLAST reside on a remote server at EBI. Figure 1 (left) shows a schematic representation of this workflow as it would be implemented in Taverna. Here the parameters of both tools are explicitly represented as nodes and a link between the parameter nodes and the programs are extended. Figure 2 shows the corresponding SCUFL file, which is generated from Taverna after the user composes this workflow in the Taverna authoring (workbench) module. In this figure, the input of seqret is defined by a data source processor (Line 1). The seqret processor itself is defined in Lines 2-5. The only parameter of the seqret program is the specification of the output format, which is defined by the string constant processor in Lines 6-8. seqret has two incident links: The first passes the input sequence (Line 9), and the second passes the parameter (Line 10). The output of seqret is passed to the BLAST node by the link in Line 11. Note that the names of the program input and parameter (e.g., "blast:query") are defined by the tool/service and are used as explained in the interface/manual. The processor defining the BLAST program is defined in Lines 12-15. There are two BLAST parameters in use: The first is the type of database; this is defined by the processor in Lines 16-18 and passed to BLAST node by the link in Line 19. The second is the output format, which is defined by the processor in Lines 20-22 and passed to BLAST node by the link in Line 23. The output of BLAST is received by one data sink node (Lines 24,25).

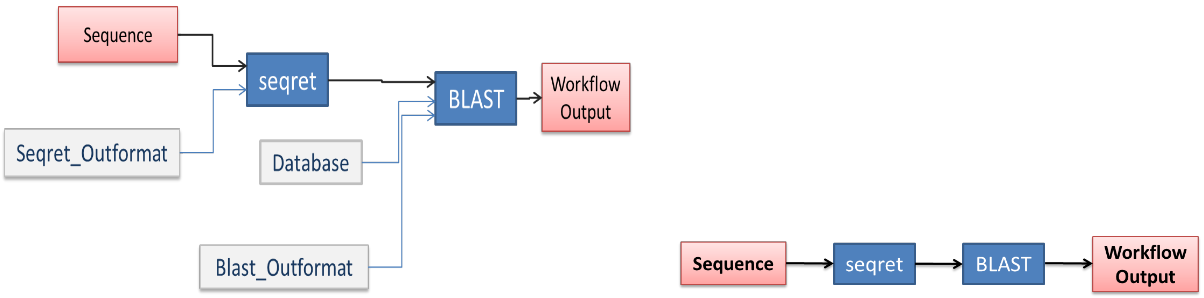

Figure 1: Left: The schematic representation of an example workflow as implemented in Taverna. Dark lines refers to input/output data flow. Light lines refers to passing parameters values. Source and sink nodes are the "Sequence" and "Workflow Output" nodes. Right: The schematic representation of the same workflow as implemented in Tavaxy. Tool parameters are defined implicitly as attributes within the processor node.

Taverna

```
<s:source name="Sequence" />
<s:processor name="seqret">
  <s:description>Reads and writes (returns) sequences</s:description>
   <s:soaplabwsdl>http://www.ebi.ac.uk/soaplab/services/edit.seqret</s:soaplabwsdl>
</s:processor>
<s:processor name="seqret_out_format" boring="true">
  <s:stringconstant>fasta</s:stringconstant>
</s:processor>
<s:link source="Sequence" sink="seqret:sequence_direct_data" />
<s:link source="seqret_out_format:value" sink="seqret:osformat_outseq" />
<s:link source="seqret:outseq" sink="blast:query" />
<s:processor name="blast">
  <s:description>Run Blast Query</s:description>
 <s:soaplabwsdl>http://www.ebi.ac.uk/soaplab/services/blastn</s:soaplabwsdl>
</s:processor>
<s:processor name="database" boring="true">
  <s:stringconstant>nt</s:stringconstant>
</s:processor>
<s:link source="database:value" sink="blast:database" />
<s:processor name="blast_out_format" boring="true">
  <s:stringconstant>tabular</s:stringconstant>
</s:processor>
<s:link source="blast_out_format:value" sink="blast:out_fmt" />
<s:sink="workflow_output" />
<s:link source="blast:output:value" sink="workflow_output" />
```

Figure 2: Taverna implementation of the workflow in Figure 1.

1.2 Tavaxy language

Tavaxy format is tSCUFL, which is a modified version of SCUFL. I.e., it is also XML-based file format that encodes the DAG corresponding to the input workflow. In tSCUFL, the words *processor* and *link* have the same meaning as in SCUFL. The syntax of processors in tSCUFL is similar to that in SCUFL. The only difference is that the tool parameters in SCUFL are passed as input, but in tSCUFL the parameters are attributes of the processor and part of the tool definition in the system. (Each tool in Tavaxy is defined through an XML-file, where its input types and parameters are specified. More information are available in Tavaxy manual.) The data links in Tavaxy have the same syntax as in Taverna.

Tavaxy includes equivalent control flow constructs to those in Taverna. These include dependency links, conditionals, and iterations. But the constructs of Tavaxy are introduced to the user in an explicit manner, as will be explained in the following sections.

2 Translating processors and links

Depending on user choices and available local tools, processors in Taverna can be translated to equivalent ones in Tavaxy according to one of two modes: 1) Translation with replacing the involved processor tool (which is either a remote call or specific Taverna scripts) with the equivalent local tool in Tavaxy. 2) Translation without replacement. In the latter case, a sub-workflow encapsulating the non-replaced tool is generated in Taverna format. This sub-workflow is included as a node in Tavaxy and it is invoked using the Taverna engine in the run time.

2.1 Translation with replacement

Figure 3 shows an example of the seqret tool in Taverna and Tavaxy. The remote call of seqret is replaced with the local seqret tool in Tavaxy. The data links have the same format. The equivalent processors in Tavaxy are shown in the figure with the parameters included as attributes of the processor XML node.

Taverna

```
1.<s:processor name="seqret">
2. <s:description>Reads and writes (returns) sequences</s:description>
3.  <s:soaplabwsdl>http://www.ebi.ac.uk/soaplab/services/edit.seqret</s:soaplabwsdl>
4.</s:processor>
5.<s:processor name="out_format" boring="true">
6. <s:stringconstant>fasta</s:stringconstant>
7.</s:processor>
8.<s:link source="sequence" sink="seqret:sequence_direct_data" />
9.<s:link source="out_format:value" sink="seqret:osformat_outseq" />
```

Tavaxy

```
1.<s:processor name="seqret" id="EMBOSS: seqret84">
2. <s:parameter name="out_format1">fasta</s:parameter>
3.</s:processor>
4.<s:link source="sequence" sink="seqret:input1"/>
```

Figure 3: Translation of a processor in SCUFL to the equivalent one in Tavaxy. Here, the remote tool seqret is replaced by the equivalent local tool.

2.2 Translation without replacement

Processors that are not mapped to corresponding processors in Tavaxy are encapsulated in separate Taverna sub-workflows. These workflows are then executed inside Taverna by calling the Taverna workflow engine from inside Tavaxy. Therefore, a Tavaxy processor node is created in the tSCUFL file: This processor includes the Taverna sub-workflow as input and a special Tavaxy program (called tavernaRunner) that invokes the Taverna engine to execute the sub-workflow. In the translation, the input and output channels between this sub-workflow and the other workflow processors are mapped appropriately.

Figure 4 shows an example of a BLAST processor in Taverna. The translation is done at two steps: First, this processor is encapsulated in a sub-workflow. Second, a processor is added to the tSCUFL file with this sub-workflow passed as input through the parameter field.

2.3 Rewriting rules for processors and links

We write $P^n(name, description, tool)$ to denote a string that specifies a processor in Taverna. This string is the one in Figure 3 (Taverna SCUFL, Lines 1-4), where $name = seqret$ and $tool = seqret$. We write $S^n(name, string)$ to denote a string constant or a data source processor, where *name* is an identifier, and *string* is the included string/input. Let $P^x(name, description, tool)$ be the string describing a processor in Tavaxy, as given in Figure 3 (Tavaxy tSCUFL, Lines 1-3). Let $L(source\_node, sink\_node)$ denote a link connecting a source node to a sink node. The re-writing rules for translating processors and links from SCUFL to tSCUFL are stated in the table of Figure 5.

In the processor rule, the processor and all incident links connecting it to its parameters are transformed to a processor in tSCUFL. But the parameters are considered as attributes. This rewriting rule is applied for all processors in the SCUFL file. The data links in Taverna and Tavaxy have the same syntax. Dependency links are mapped in a similar way to the data link. By applying these rules to map all processors and links, the DAG graph representing any workflow including no control constructs (i.e., data-flow oriented workflow) can be reconstructed in the tSCUFL language of Tavaxy. In the remaining parts of this document, we will discuss the translation of the three control constructs of Taverna addressed in the introduction.

3 Translating dependency links

The dependency links (also called coordination links) in Taverna are mapped to usual data links in Tavaxy, where 'dummy' data is passed between the respective nodes. Figure 6 shows the SCUFL representation of a dependency link using the co-ordination element and the corresponding representation of the same functionality in Tavaxy. The rewriting tool to this node is similar to that of the data link, once the dependency (coordination) node in the XML file is recognized.

4 Translating conditionals

Figure 7 shows the schematic represntation of the conditional *if-else* construct in both Taverna and Tavaxy. As shown in the figure, this construct is not explicitly represented in Taverna. It requires that the workflow designer use the two *failure* nodes known as FAIL_IF_TRUE and FAIL_IF_FALSE. (These are special built-in scripts in Taverna.) The user has to support a Java code (beanshell script) to check for certain condition. This script passes a boolean value to the FAIL_IF_FALSE and FAIL_IF_TRUE nodes. Dependency links are used to connect the failure nodes and the dependent SCUFL nodes.

In Tavaxy, the structure of the conditional construct is similar. It however does not use failure links and have explicit *if-else* (*switch*) node. In tSCUFL, the user has also to specify a script to check for a certain condition.

Figure 8 shows the SCUFL and tSCUFL representation corresponding to the conditionals in Figure 7.

4.1 Writing rule for translating conditionals

As can be seen in the example, the *if-else* construct in Taverna is composed of the following four components:

1. A processor including a script that checks a certain condition. The output of this processor goes to two failure processors. We write $CS^v(name, description, script)$ to denote this processor, where *name*, *description*, and *script* refer to its name, description, and the script code (either built-in or referred to by a file), respectively.

2. Two failure processors (FAIL_IF_TRUE and FAIL_IF_FALSE). We write $FT()$ and $FF()$ to denote these two processors.

3. Two data links connecting the check condition processor with the failure processors. We write $L(source, sink)$ to denote such a link.

4. Two dependency links connecting the failure nodes to two other nodes in the workflow. We write $L_D(source, sink)$ to denote such a dependency link.

In Tavaxy, the structure of the conditional construct is similar and it is composed of the following four components:

1. A processor including a script that checks a certain condition. The output of this processor goes to the switch processor. We write $CS^x(name, description, script)$ to denote this processor, where *name*, *description*, and *script* refers to its name, description, and the script code (either built-in or refered to by a file).

2. *Switch* processor, which receives an input from the CS and accordingly sends a failure token to one of its two output branches. The default is that if it receives 'TRUE', a success token (or real data) is send on the first branch and a failure token on the second. However, this behavior can be modified by the user by adjusting the script associated with this processor. (See the manual for more details.) We write $SWITCH^x(script)$ to denote a switch processor.

3. One link connecting the processor that checks the condition to the switch processor.

4. Two links connecting the switch processor to the succeeding nodes in the workflow.

The table in Figure 5 contains the re-writing rule for mapping each conditional construct in Taverna to the equivalent one in Tavaxy. In the mapping, the CS script is reused. The FF and FT as well as the associated links are no longer needed, because the switch node implicitly takes care of the involved conditional logic.

5 Translating iterations

Tavenra and Tavaxy iteration constructs are very similar to each other. Both execute a sub-workflow iteratively until some condition is satisfied. The difference is that the iteration construct in SCUFL is not explicit. It requires that the workflow designer use the RETRY and failure (FAIL_IF_FALSE, FAIL_IF_TRUE) methods. Figure 9 shows the schematic representation of the iteration construct in both Taverna and Tavaxy. The user has to support a Java code (beanshell script) to check for certain condition. This script passes a boolean value to the FAIL_IF_FALSE and FAIL_IF_TRUE nodes.

Figure 10 shows the representation of the iteration construct in both systems. In SCUFL and tSCUFL, the iteration construct runs a sub-workflow processor in an iterative manner. This sub-workflow contains a processor for checking the condition and producing a boolean value. The boolean value is passed to a processor that fails, leading to non-execution of the successor tasks.

5.1 Writing rule for translating iterations

As can be seen in the example, the *iteration* construct in Taverna is composed of the following three main parts:

1. A processor encapsulating the iteration construct.

2. An embedded (sub-) workflow with the directives 'maxretries' and 'retrydelay' to iterate this workflow maximum x iterations AND as long as a condition fails, where x is defined by the user.

3. A processor embedded in the iteration sub-workflow including a script that checks a certain condition. The output of this processor goes to a failure processor, defined in the next item.

4. Failure node (FAIL_IF_FALSE or FAIL_IF_TRUE) to decide if continue iteration or break. We write CS to denote this script.

We write $I^v(W_i^v, CS, F)$ to denote an iteration processor in SCUFL with the embedded (sub-) workflow W_i, the conditional script CS, and the failure function F.

Tavaxy includes the four items except for the failure links. Hence, the iteration pattern in tSCUFL is similarly denoted by $I^x(W_i^x, CS)$. So, the re-writing rule is simple, once we mapped the sub-workflows $W^v \rightarrow W^x$ based on the discussion in previous sections. The table in Figure 5 contains the re-writing rule for mapping each iteration construct in Taverna to the equivalent one in Tavaxy.

Note: The 'maxretries' and 'retrydelay' parameters are needed by Taverna to recall a remote web-services in the case of failure, which is common in distributed settings. In the design of Tavaxy conditionals we did not see a demanding need for using these parameters explicitly, because of two reasons: 1) Tavaxy runs on local infrastructure, and 2) the user can include these state information in the CS script associated with this construct.

Taverna workflow with remote BLAST

```
<s:processor name="blast">
  <s:description>Execute Blast</s:description>
  <s:arbitrarywsdl>
    <s:wsdl>http://xml.nig.ac.jp/wsdl/Blast.wsdl</s:wsdl>
    <s:operation>searchSimple</s:operation>
  </s:arbitrarywsdl>
</s:processor>
<s:link source="database:value" sink="blast:database" />
<s:link source="blast_out_format:value" sink="blast:out_fmt" />
<s:link source="sequence"sink="blast:query" />
<s:link source="blast:Result" sink="next_node" />
```

Encapsulated sub-workflow

```
<s:source name="source1"/>
<s:source name="source2"/>
<s:source name="source3"/>
<s:sink   name="sink1"/>
<s:processor name="blast">
  <s:description>Execute Blast</s:description>
  <s:arbitrarywsdl>
    <s:wsdl>http://xml.nig.ac.jp/wsdl/Blast.wsdl</s:wsdl>
    <s:operation>searchSimple</s:operation>
  </s:arbitrarywsdl>
</s:processor>
<s:link source="source1" sink="blast:database" />
<s:link source="source2" sink="blast:out_fmt" />
<s:link source="source3" sink="blast:query" />
<s:link source="blast:Result" sink="sink1" />
```

Invocation of the encapsulated workflow in Tavaxy

```
<s:processor name="blast" id="tavernaRunner">
  <s:parameter name="inputs_in_json">
  {"input1":"source1","input2":"source2","input3":"source3"}
  </s:parameter>
  <s:parameter name="outputs_in_sjson">
  {"output1":"sink1"}
  </s:parameter>
  <s:parameter name="workflowPath">SCUFL file</s:parameter>
</s:processor>
<s:link source="database:value" sink="blast:input1"/>
<s:link source="blast_out_format:value" sink="blast:input2"/>
<s:link source="sequence"  sink="blast:input3"/>
<s:link source="blast:output1" sink="next_node"/>
```

Figure 4: Translation of Tavaxy sub-workflows without tool replacement. "prev_node" and "next_node" are preceding and succeeding nodes connected to the BLAST processors. The three sources in the intermediate sub-workflow are associated with the three inputs in the Tavaxy processor that executes this sub-workflow.

Rule 1: Processors	$\left\{\begin{array}{c} P^v(N,D,X), \\ S^v(N_{i_1},s_{i_1}), \\ ..., \\ S^v(N_{i_j},st_{i_j}), \\ L(S^v(N_{i_1},s_{i_1}),P^v(N,D,X)), \\ ..., \\ L(S^v(N_{i_j},s_{i_j}),P^v(N,D,X)), \end{array}\right\} \rightarrow P^x(N,D,X,s_{i_1},...,s_{i_j})$
Rule 2: Data sources	$D(source\_data) \rightarrow D(source\_data)$
Rule 3: Data sinks	$D(sink\_data) \rightarrow < STDOUT >$
Rule 4: Links	$L(P_i^v(N_i,D_i,X_i),P_j^v(N_j,D_j,X_j)) \rightarrow L(P_i^x(N_i,D_i,X_i),P_j^x(N_j,D_j,X_j))$
Rule 5: Conditionals	$\left\{\begin{array}{c} CS^v(name,description,script), \\ FT, \\ FF, \\ L(CS^v,FT), \\ L(CS^v,FF), \\ L_D(FT,P_i^v), \\ L_D(FT,P_j^v) \end{array}\right\} \rightarrow \left\{\begin{array}{c} CS^x(name,description,script), \\ SWITCH^x(script), \\ L(CS^x,script), \\ L(SWITCH^x,P_i^x), \\ L(SWITCH^x,P_j^x) \end{array}\right\}$
Rule 6: Iterations	$I^v(W_i^v,CS,F) \rightarrow I^x(W_i^x,CS)$

Figure 5: Re-writing rules for translating processors, data sources, data sinks, links, conditionals, and iterations from SCUFL to tSCUFL.

Taverna

```
<s:coordination name="Dependency_Link1">
<s:condition>
  <s:state>Completed</s:state>
   <s:target>Task1</s:target> /* source task */
 </s:condition>
 <s:action>
<s:target>Task2</s:target> /* sink task */
 <s:statechange>
      <s:from>Scheduled</s:from>
         <s:to>Running</s:to>
  </s:statechange>
</s:action>
</s:coordination>
```

Taverna

```
<s:link source="Task1:dummy" sink"Task2:dummy" />
```

Figure 6: Dependencies between two processors 'Task1' and 'Task2'.

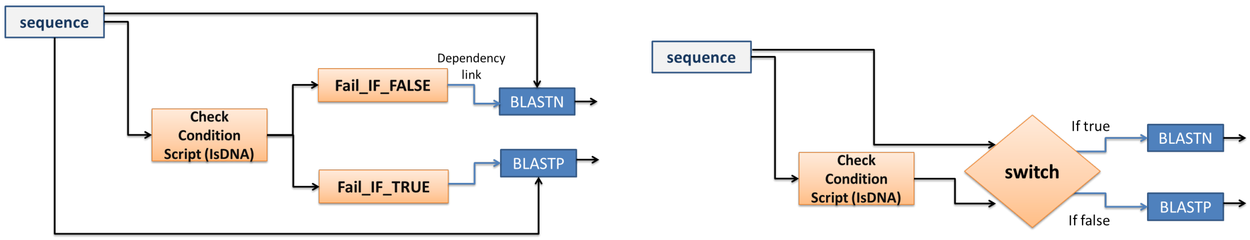

Figure 7: Schematic representation of the conditional construct in Taverna (left), and in Tavaxy (right).

Taverna

```
<s:processor name="IsDNA">
   <s:beanshell>
     <s:scriptvalue> /* Java code (Beanshell)  that checks if sequence is a DNA*/ ..
     <s:beanshellinputlist>
       <s:beanshellinput s:syntactictype=""text/plain"">sequence</s:beanshellinput>
     </s:beanshellinputlist>
     <s:beanshelloutputlist>
       <s:beanshelloutput s:syntactictype=""text/plain"">BooleanValue</s:beanshelloutput>
     </s:beanshelloutputlist>
      <s:dependencies s:classloader="iteration" />
    </s:beanshell>
</s:processor>
<s:processor name="Fail_if_false">
   <s:local>org.embl.ebi.escience.scuflworkers.java.FailIfFalse</s:local>
</s:processor>
<s:processor name="Fail_if_True">
   <s:local>org.embl.ebi.escience.scuflworkers.java.FailIfTrue</s:local>
</s:processor>
<s:coordination name="Depend_On_Fail_if_true">
    <s:condition>
      <s:state>Completed</s:state>
      <s:target>Fail_if_true</s:target>
    </s:condition>
    <s:action>
      <s:target>Run_BLASTP</s:target> /*Run BLASTP program as the sequence is protein*/
      <s:statechange>
        <s:from>Scheduled</s:from>
        <s:to>Running</s:to>
      </s:statechange>
    </s:action>
</s:coordination>
<s:coordination name="Depend_On_Fail_if_false">
    <s:condition>
      <s:state>Completed</s:state>
      <s:target>Fail_if_false</s:target>
    </s:condition>
    <s:action>
      <s:target> Run_BLASTN</s:target>/*Run BLASTN program as the sequence is DNA*/
      <s:statechange>
        <s:from>Scheduled</s:from>
        <s:to>Running</s:to>
      </s:statechange>
    </s:action>
</s:coordination>
<s:link source="IsDNA:BooleanValue" sink="Fail_if_false:test" />
<s:link source="IsDNA:BooleanValue" sink="Fail_if_true:test" />
```

Tavaxy

```
1.<s:processor name="IsDNA" id="beanshellRunner">
Name of Java program (Beanshell) that checks if the sequence is DNA
</s:processor>
2.<s:processor id="swtich" name="swtich0">
3. <s:parameter name="script">switch_script</s:parameter> /* to direct the output*/
4.</s:processor>
5.<s:link source="IsDNA:output" sink="swtich0:condition"/>
6.<s:link source="swtich0:falseOut" sink="Run_BLASTP" />
7.<s:link source="swtich0:trueOut" sink="Run_BLASTN" />
```

Figure 8: Example of the *if-else* construct in SCUFL and the corresponding switch pattern in Tavaxy.

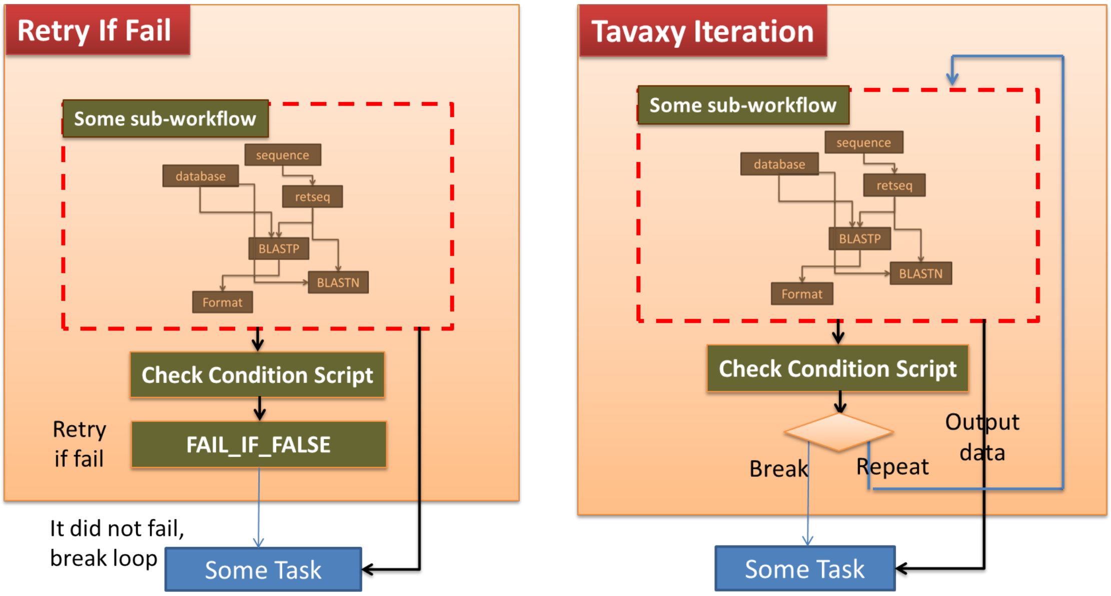

Figure 9: Schematic representation of the iteration construct in Taverna (left), and Tavaxy (right).

Taverna

```
<s:processor name="iteration">
  <s:workflow maxretries="10" retrydelay="1000">
    <s:scufl version="0.2" log="0">
      <s:workflowdescription sub_workflow_inserted_here /s:workflowdescription>
      <s:processor name="check_condition"> Check_Condition_Script</s:processor>
      <s:processor name="Fail_if_false">
        <s:local critical="true">org.embl.ebi.escience.scuflworkers.java.FailIfFalse... </s>
      </s:processor>
      <s:link source="check_condition:value" sink="Fail_if_false:test" />
    </s:scufl>
  </s:workflow>
</s:processor>
```

Tavaxy

```
<s:processor name="iteration" id="iteration">
    <s:scufl version="0.2" log="0">
      <s:workflowdescription sub_workflow_inserted_here .../s:workflowdescription>
      <s:processor name="check_condition" id="...">Check_Condition_Script</processor>
    </s:scufl>
   <s:properties>
      <s:condition>
         <port>check_condition|Output</port>
         <operator>Equals</operator>
         <value>false</value>
      </s:condition>
    </s:properties>
  </s:processor>
```

Figure 10: The iteration pattern in SCUFL and tSCUFL.
