# Supplementary figures and images for: Tavaxy: Integrating Taverna and Galaxy workflows with cloud computing support
Source: BMC Bioinformatics. 2012 May 4;13:77. doi: 10.1186/1471-2105-13-77 (PMC3583125; doi:10.1186/1471-2105-13-77)

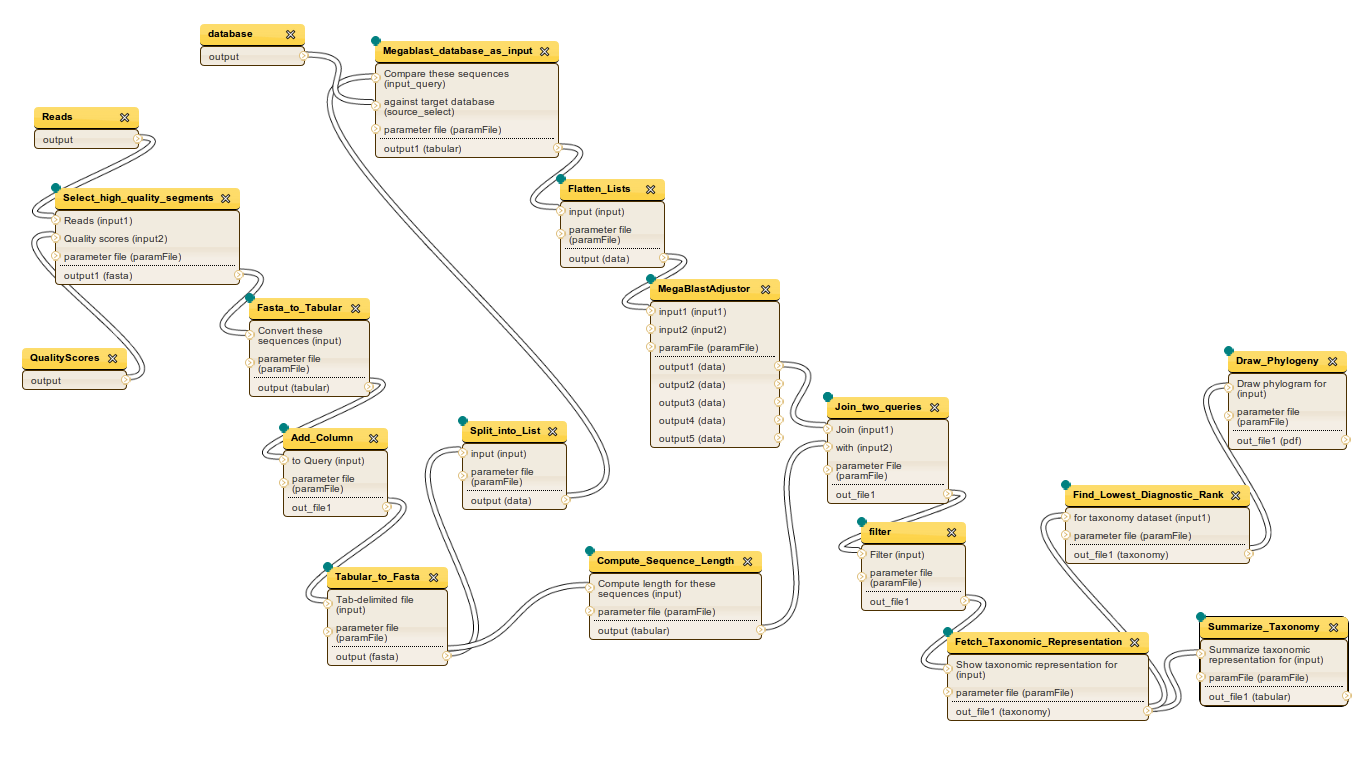

Supplement: Additional file 2 — Paper figures in original size. Compressed folder containing the paper figures in original size for better visualization. [file 1471-2105-13-77-S2.zip › SubmissionFigures/Fig10MetaGenomicsTavaxy.png]

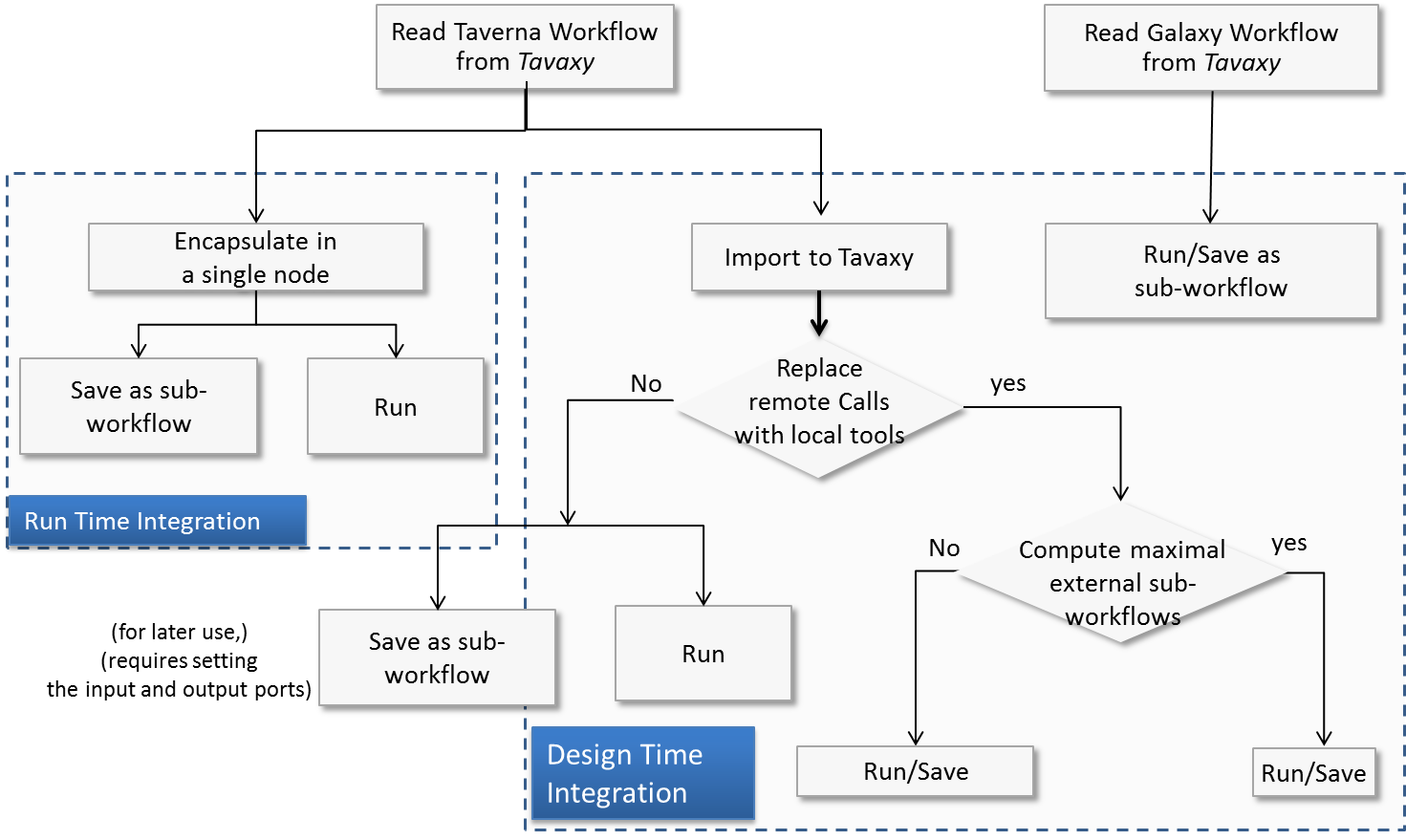

Supplement: Additional file 2 — Paper figures in original size. Compressed folder containing the paper figures in original size for better visualization. [file 1471-2105-13-77-S2.zip › SubmissionFigures/Fig1TavaxyUseDiagram.png]

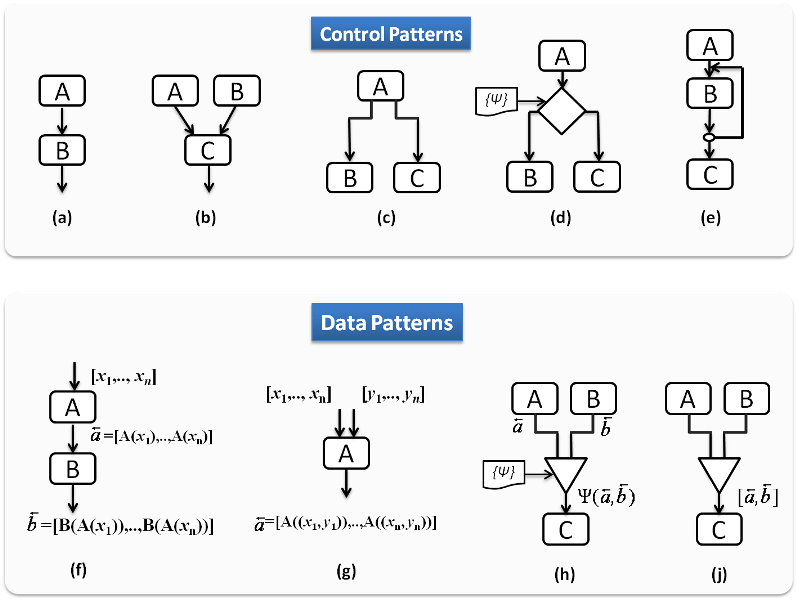

Supplement: Additional file 2 — Paper figures in original size. Compressed folder containing the paper figures in original size for better visualization. [file 1471-2105-13-77-S2.zip › SubmissionFigures/Fig2WFPatterns.png]

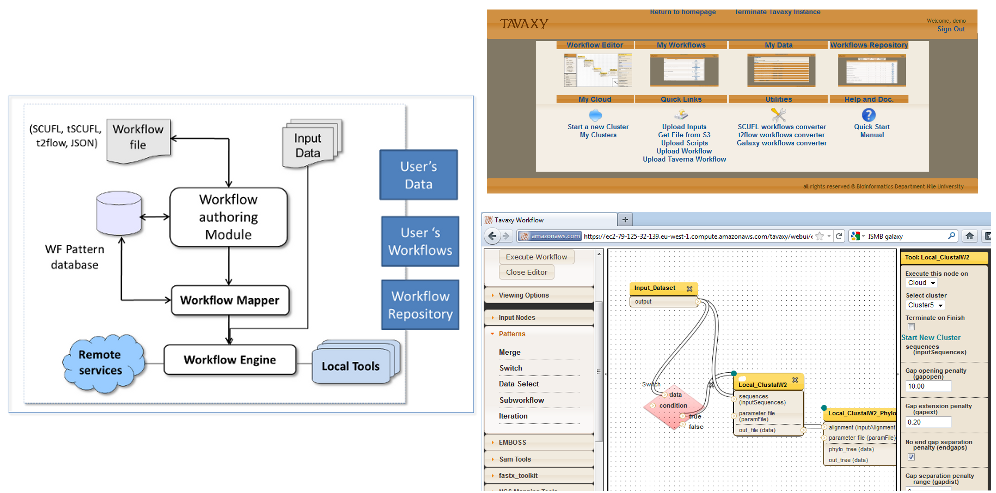

Supplement: Additional file 2 — Paper figures in original size. Compressed folder containing the paper figures in original size for better visualization. [file 1471-2105-13-77-S2.zip › SubmissionFigures/Fig3Tavaxy2Architecture.png]

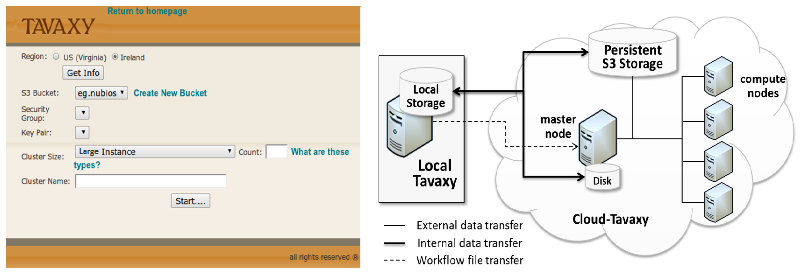

Supplement: Additional file 2 — Paper figures in original size. Compressed folder containing the paper figures in original size for better visualization. [file 1471-2105-13-77-S2.zip › SubmissionFigures/Fig4CloudTavaxyArchitecture.png]

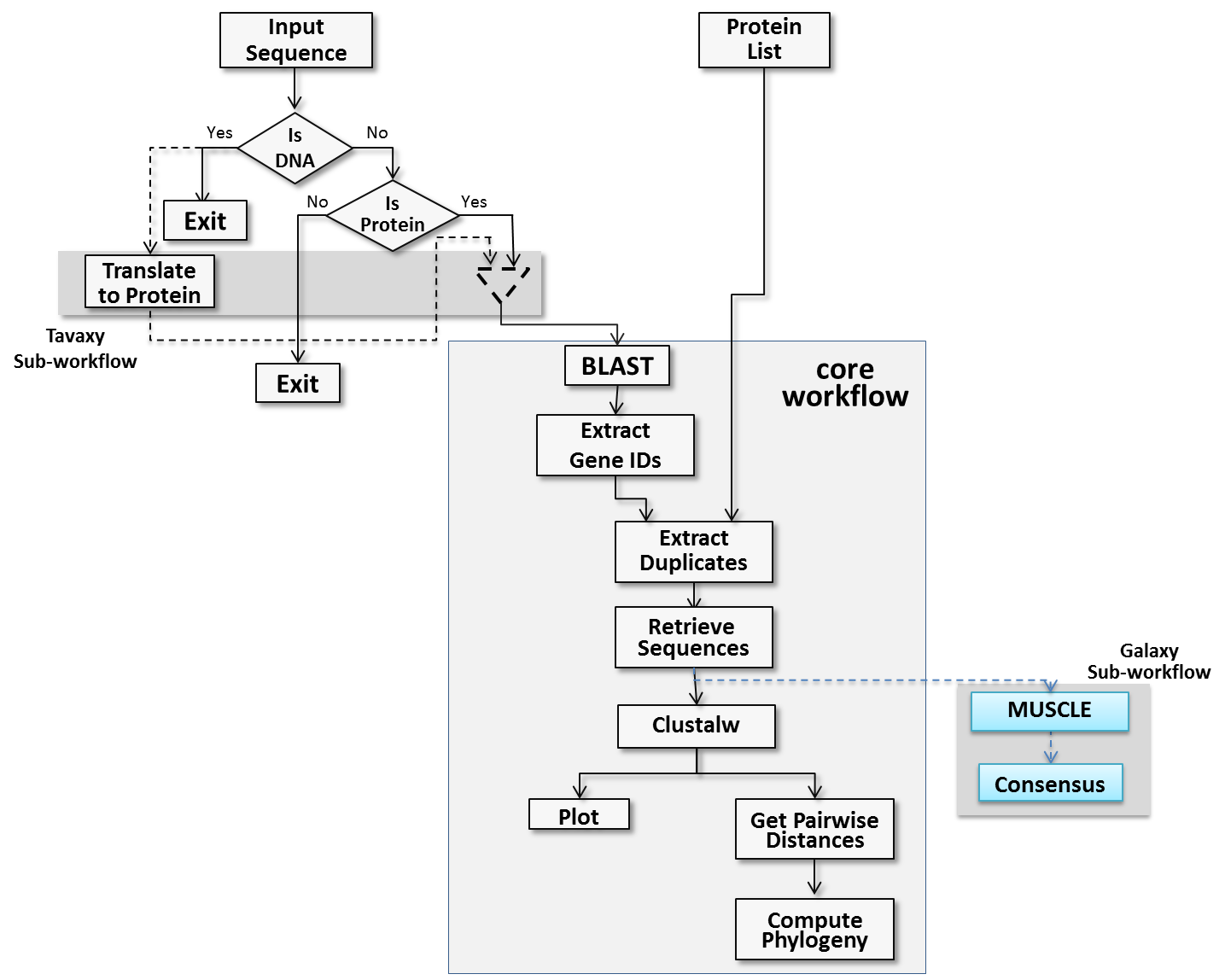

Supplement: Additional file 2 — Paper figures in original size. Compressed folder containing the paper figures in original size for better visualization. [file 1471-2105-13-77-S2.zip › SubmissionFigures/Fig5HomologyWF2.png]

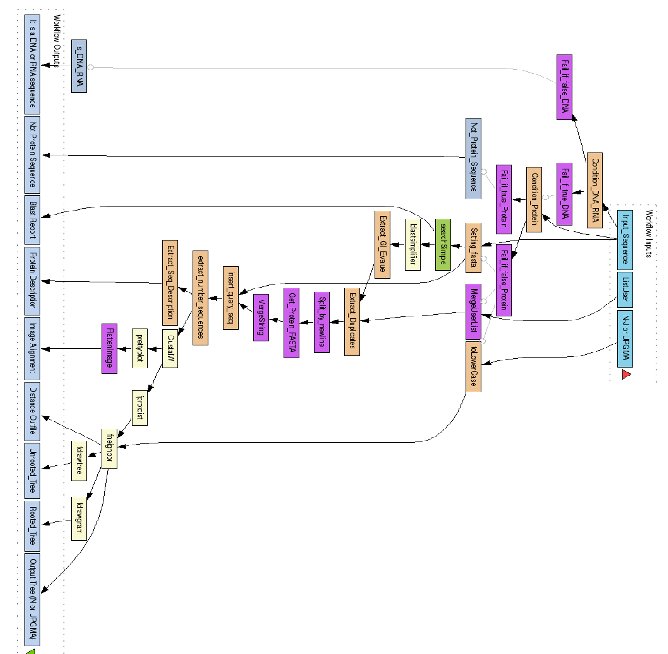

Supplement: Additional file 2 — Paper figures in original size. Compressed folder containing the paper figures in original size for better visualization. [file 1471-2105-13-77-S2.zip › SubmissionFigures/Fig6WFprotana5539.png]

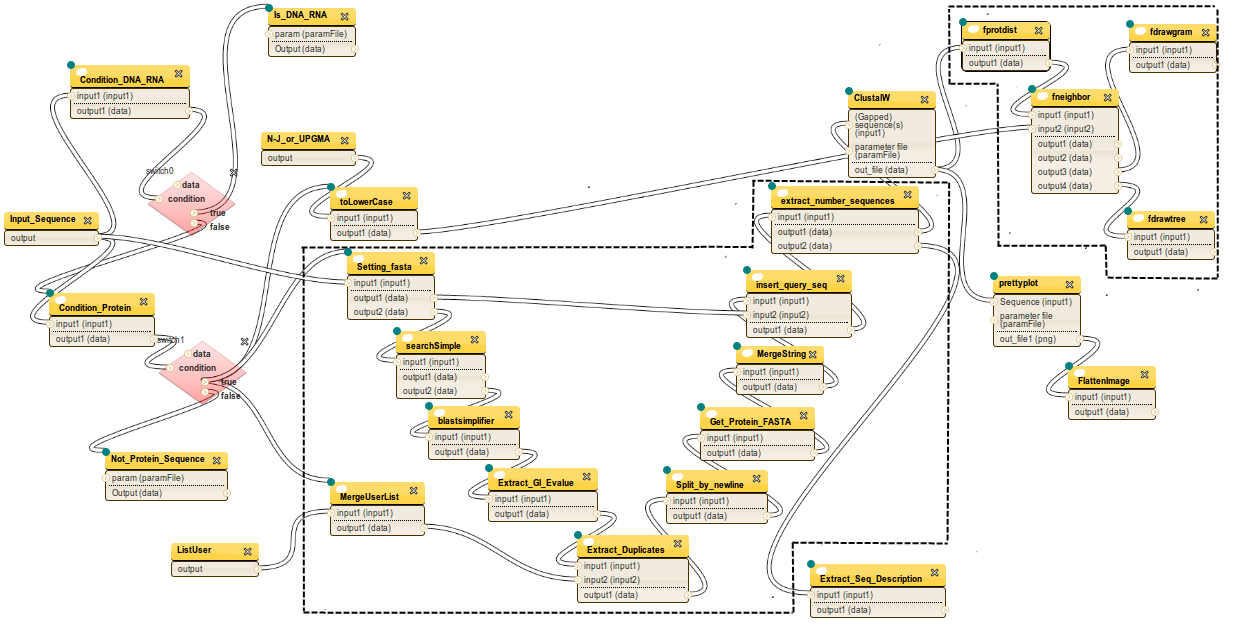

Supplement: Additional file 2 — Paper figures in original size. Compressed folder containing the paper figures in original size for better visualization. [file 1471-2105-13-77-S2.zip › SubmissionFigures/Fig7HomologyTavaxyNon-optimized2.png]

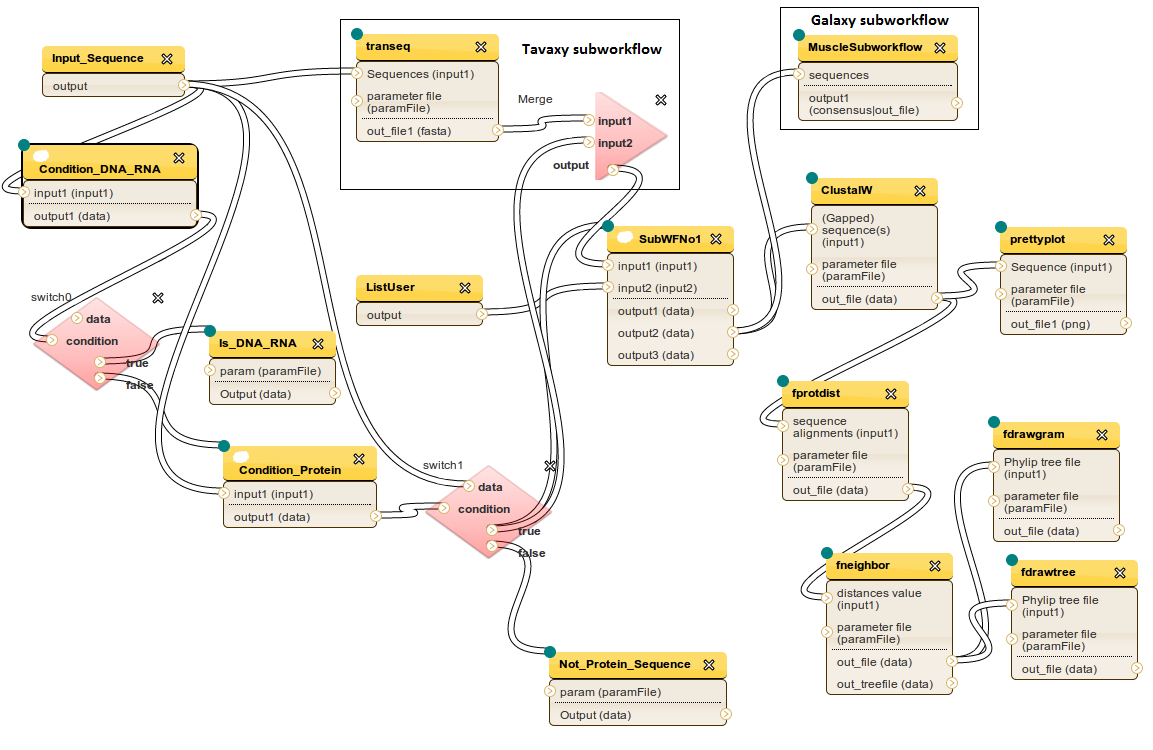

Supplement: Additional file 2 — Paper figures in original size. Compressed folder containing the paper figures in original size for better visualization. [file 1471-2105-13-77-S2.zip › SubmissionFigures/Fig8HomologyWFOptimizedWithMuscle.png]

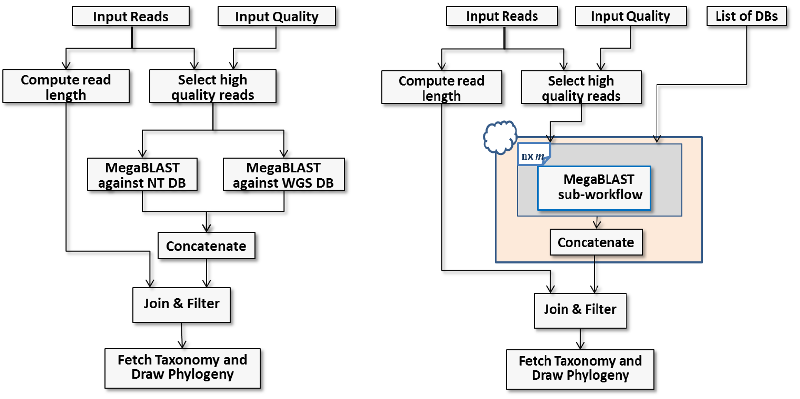

Supplement: Additional file 2 — Paper figures in original size. Compressed folder containing the paper figures in original size for better visualization. [file 1471-2105-13-77-S2.zip › SubmissionFigures/Fig9MetaWFGalaxyTavaxy.png]
